# A novel RNA sequencing data analysis method for cell line authentication

**DOI:** 10.1371/journal.pone.0171435

**Published:** 2017-02-13

**Authors:** Erik Fasterius, Cinzia Raso, Susan Kennedy, Nora Rauch, Pär Lundin, Walter Kolch, Mathias Uhlén, Cristina Al-Khalili Szigyarto

**Affiliations:** 1 School of Biotechnology, Royal Institute of Technology, Stockholm, Sweden; 2 Systems Biology Ireland, University College Dublin, Belfield, Dublin 4, Ireland; 3 Science for Life Laboratory, Dept of Biochemistry and Biophysics, Stockholm University, Stockholm, Sweden; 4 Conway Institute of Biomolecular & Biomedical Research, University College Dublin, Belfield, Dublin 4, Ireland; 5 School of Medicine, University College Dublin, Belfield, Dublin 4, Ireland; 6 Science for Life Laboratory, Stockholm, Sweden; New York University School of Medicine, UNITED STATES

## Abstract

We have developed a novel analysis method that can interrogate the authenticity of biological samples used for generation of transcriptome profiles in public data repositories. The method uses RNA sequencing information to reveal mutations in expressed transcripts and subsequently confirms the identity of analysed cells by comparison with publicly available cell-specific mutational profiles. Cell lines constitute key model systems widely used within cancer research, but their identity needs to be confirmed in order to minimise the influence of cell contaminations and genetic drift on the analysis. Using both public and novel data, we demonstrate the use of RNA-sequencing data analysis for cell line authentication by examining the validity of COLO205, DLD1, HCT15, HCT116, HKE3, HT29 and RKO colorectal cancer cell lines. We successfully authenticate the studied cell lines and validate previous reports indicating that DLD1 and HCT15 are synonymous. We also show that the analysed HKE3 cells harbour an unexpected KRAS-G13D mutation and confirm that this cell line is a genuine KRAS dosage mutant, rather than a true isogenic derivative of HCT116 expressing only the wild type KRAS. This authentication method could be used to revisit the numerous cell line based RNA sequencing experiments available in public data repositories, analyse new experiments where whole genome sequencing is not available, as well as facilitate comparisons of data from different experiments, platforms and laboratories.

## Introduction

The prevalence of using human cell lines as *in vitro* model systems for cancer research is due to their ability to replace scarce and valuable human samples. Cell lines offer an unlimited source of biological material and represent homogeneous cell type populations, which facilitates both experimental procedures and interpretation of results in comparison to the analysis of tissues and organs. They are also easy to use since well-developed protocols are available for culturing, genetic manipulation, molecular analysis and other assay-based experiments. Cell lines offers a cost-effective source of materials that bypasses ethical concerns raised by the use of other biological material like human or animal tissues. Using cell lines to model human biology, test efficacy of therapies and produce therapeutic proteins is common practice in research, yet it is widely acknowledged that contamination of said cell lines is a prevalent problem. [1, 2] Mycoplasma contamination frequently occurs during cultivation of cell lines and is also present in many cell banks and repositories, but can be tested for and eliminated with proper culturing techniques. [3] Common contaminants are other human cell lines, such as HeLa, but it has also become increasingly apparent that many cell lines become cross-contaminated at their creation. [4] Cross-species contamination is less of a problem than the ubiquitous intra-species contamination, but should not be neglected. Genetic drift and other subculturing effects can also affect the cell lines’ suitability as an experimental model system, and long-time culturing should thus be avoided. [5]

The awareness of pitfalls related to cell line authenticity has increased rapidly since 2007. [6] The analysis of Short Tandem Repeats (STRs) across several loci has become the standard recommended by the American Type Culture Collection (ATCC) and the American National Standards Institute (ANSI). [7] Another increasingly common method is Single Nucleotide Polymorphism/Variant (SNP/SNV) genotyping. [8] Using SNV genotyping rather than STR profiling can alleviate some of the problems, such as microsatellite instability, but a greater degree of certainty can be achieved by combining both methods. [9] While STR and SNV-based approaches are well-supported by already existing human cell line profiles, that is usually not the case for other species. There are, however, PCR-based methods available to identify cross-species contamination. [10] Besides the immediate need for cell authentication procedures when initiating new studies, data from already performed experiments remain difficult to compare if the authenticity of the cells used is inadequate. Between 15% to 20% of the cells currently in use have been shown to be misidentified, including a large number of datasets stored in public repositories. [11] Freedman *et al*. has emphasised the need for new and robust methods for cell line authentication. [12]

With the advent of high-throughput sequencing technologies such as RNA sequencing (RNA-seq), new venues for exploiting large amounts of transcript data have become available. [13] RNA-seq has been widely utilised to characterise the transcript landscape of tissues and cells in the absence and presence of stimuli. Additionally, studies have shown the potential and feasibility of using RNA-seq data to find both SNVs in expressed transcripts [14] and cross-species contaminations [3], opening up new possibilities for cell line authentication.

In the present study, we present a pipeline utilising RNA-seq data analysis in combination with already existing mutational profiles for human cell line authentication. We show that comparing RNA-seq data from several colorectal cancer cell lines (COLO205, DLD1, HCT15, HCT116, HKE3, HT29 and RKO) to databases such as the *Catalogue of somatic mutations in cancer* (COSMIC) [15] can authenticate cell lines to a high degree of certainty, give in-depth information about errors in known variants as well as point to possible HeLa contaminations. As the availability of RNA-seq experiments and data repositories continues to increase, so does the opportunity of using this data for more reliable and large-scale cell line authentication efforts.

## Materials and methods

### Cell lines

Seven colorectal cancer cell lines, COLO205, DLD1, HCT15, HCT116, HKE3, HT29 and RKO (with two different datasets for HCT116), were analysed in the study. HCT116a, HKE3 and RKO were analysed using data obtained from in-house culturing and sequencing. The data for COLO205, HCT116b, HCT15 and HT29 was downloaded from the Gene Expression Omnibus (GEO) database [16] under the accession number GSE73318 [17] as SRA files and converted to FASTQ using *fastq-dump* from the *SRA toolkit*. [18] The DLD1 data was similarly downloaded and processed from GEO accession GSE75189. [19]

### Cell line cultivations and RNA extractions

The HCT116a and RKO cell lines were cultivated in standard McCoy medium with 10% Foetal Bovine Serum and were split 1:5 every third or fourth day (Sigma Aldrich). The HKE3 cell line was similarly cultivated in standard DMEM media with 10% FBS. RNA extractions were performed at 80% confluence with the *RNeasy Plus Mini Kit* (Qiagen) as per the manufacturer’s instructions with three replicates each for HCT116/RKO and four replicates for HKE3. Cells were lysed directly in the dish using 600 μL buffer RLT Plus supplemented with *β*-mercaptoethanol. The extracted RNA was stored in −80°C prior to sequencing.

### Library preparation and sequencing

RNA-seq library preparation was performed with Illumina’s *TruSeq mRNA* kit with poly-A selection (200 ng RNA per sample); all samples had a RIN value of 10 as measured with the *Agilent 2100 BioAnalyzer*. Clustering was performed using the *cBot* cluster-generation system. Sequencing was performed on a *HiSeq2500* instrument with a 2x101 bp setup in HighOutput mode (HiSeq Control Software 2.0.12.0/RTA 1.17.21.3) for HCT116 and RKO, and with a 2x126 bp setup in RapidHighOutput mode for HKE3 (HiSeq Control Software 2.2.38/RTA 1.18.61). Conversion of obtained bcl files to FASTQ was performed using *bcl2FASTQ* (v1.8.3) and the Sanger / phred33 / Illumina 1.8+ quality scale from Illumina’s *CASAVA* software suite.

### Analysis of cell line authenticity

The RNA-seq read alignment to the *GRCh37* reference was performed using the *STAR* (v2.5.1b) two-pass method, where splice-junctions found in a first alignment step is used in the second. This two-pass method increases the quality of the alignment as it pertains to downstream variant calling (which uses the sequences, positions and quantity of the aligned reads) compared to single-pass methods. [20] RNA-seq variant calling was performed using the *GATK* Best Practice workflow (v3.5.0, December 2015; https://www.broadinstitute.org/gatk/guide/article?id=3891) [21] Briefly, duplicate reads were removed, followed by re-alignment of indels and re-calibration of base quality scores, in order to increase the accuracy of the subsequent variant calling using GATK’s *HaplotypeCaller*. In order to increase the coverage of the data, confident homozygous reference calls were also included. The resulting variants were filtered to have at least a *Fisher Strand* value above 30 and a *Quality by Depth* value greater than two. Clusters of at least three SNVs within a 35 base pair window were also filtered, followed by annotation with *SnpSift* and *SnpEff* (v4.2). [22, 23] Downstream filtering and analyses were performed with in-house *Python* and *R* scripts, for which detailed code is available in the supplementary information. COSMIC data from the *GRCh37* assembly [15] was used for comparisons to the RNA-seq variant calls. Read counting was performed using *featureCounts* (v1.4.5). [24] Differential expression analysis was performed with *DESeq2* (v1.6.3) [25]. Analysis of KRAS isoform expression was performed with *Kallisto* (v0.42.1). [26] The raw data for HCT116a, HKE3 and RKO as well as processed variant calling data has been uploaded to the GEO under accession number GSE81194.

### PCRs and Sanger sequencing

All PCR reactions were performed using *Dynazyme II DNA Polymerase* (Thermo Scientific) as per the manufacturer’s instructions in reaction volumes of 50 μL with 100 ng of DNA template and 1 U of Dynazyme. cDNA synthesis was performed using the *SuperScript III First Strand Synthesis System* (Thermo Scientific) with oligo(dT) as per the manufacturer’s instructions. Custom DNA primers were purchased from *Integrated DNA Technologies* and Sanger sequencing was performed by *Eurofin Genomics*. Primer sequences are listed in S1 File.

### KRAS protein quantification

DH*α* *E*. *coli* transformed with a GST-RAF-RBD construct were grown overnight at 37°C in 20 ml of LB medium containing ampicillin. The pre-culture was subsequently added to 500 ml of LB medium and grown until the OD600 was 0.6–0.8. GST-RBD expression was induced by adding IPTG at a final concentration of 1 mM, followed by incubation for 1–2 h at 37°C. The bacteria were collected by centrifugation and the pellets were frozen down at −80°C. Frozen pellets were resuspended in 10 ml of PBS (ThermoFisher Scientific) containing 1% NP40 (Calbiochem) and leupeptin (10 μg/ml, Sigma Aldrich), followed by sonication using a *Syclon Ultrasonic Homogenizer* at 8–10% power with repeated 6 s on / 1 s off cycles for a total of 5 min. Following centrifugation at 4000 rpm for 30 min (4°C), the supernatant was collected and incubated at 4°C with rotation and 500 μL of *Glutathione sepharose 4b* beads (GE Healthcare) for 3 h. The samples were centrifuged at 4000 rpm for 1 min, followed by discarding of the supernatants. Beads were resuspended in 1 ml of PBS containing 1% NP40 and aliquoted into two 1.5 ml eppendorf tubes. Beads were then subjected to 5 wash cycles: ice cold PBS containing 1% NP40 (twice), ice cold PBS (twice), and a final wash in ice cold magnesium lysis buffer (MLB: 25 mM Hepes pH 7.5, 150 mM NaCl, 1% NP40, 10% Glycerol, 25 mM NaF, 10 mM MgCl2, 1 mM EDTA, 1 mM sodium vanadate and 10 μg/ml of leupeptin). The washing supernatant was discarded, beads were resuspended in MLB buffer and stored at 4°C with protease inhibitors. Bead concentration was determined using a BSA dilution curve as a standard, running 10 μL of beads in a polyacrylamide gel followed by Coomasie staining to visualise the proteins.

HCT116 and HKE3 cells were grown on 100 mm tissue culture plates to 80% confluence. Plates were washed twice in HBS buffer and then lysed in modified MLB buffer (25 mM HEPES, pH 7.5, 150 mM NaCl, 0.5% NP40, 0.25% sodium deoxycholate, 25 mM NaF, 10 mM magnesium chloride, 1 mM EDTA, 1 mM sodium vanadate, 10 μg/ml leupeptin, 10 μg/ml aprotinin). Lysates were put on ice for 10 min with strong vortexing every 2–3 minutes, followed by centrifugation at 12000 rpm. Total protein content was measured by BCA assay (Pierce), a BSA dilution curve (measured in triplicate) and with two measurements for each sample. Cleared lysates were incubated with 15 μL of GST-RBD beads for 45 min in a rocker at 4°C, followed by washing once in full MLB buffer and four times with MLB buffer without detergent. An on-bead digestion protocol was applied as described by Turriziani *et al*. [27] followed by purification with stage tips as described by Rappsilber *et al*. [28] The tryptic peptides were analysed on an *Ultimate Ultra 3000* chromatography system coupled to a *Q-Exactive* mass spectrometer (Thermo Scientific, Germany). The sample set was analysed as four biological replicates and two technical replicates: 5 μl of tryptic digest was loaded on a homemade column (10 cm long, 75 μm inside diameter), packed with *UChrom C18* (1.8 μm) reverse phase media (nanoLCMS Solutions LCC) and then separated by a reverse phase gradient at a constant flow rate of 250 nl/min with an increasing linear gradient (2–35%) of buffer B (80% ACN, 0.5% acetic acid, total duration 65 min). The mass spectrometer was operated in positive ion mode with 2000 V potential applied to the column. Spectra were acquired through a *top 12* data-dependent tandem MS analysis mode in the range of 350–1600 m/z, selecting the 12 most intense ions for collision and acquisition. The raw data was analysed using *MaxQuant* (v1.5.0.12) with the following parameters: enzyme: trypsin; fixed modifications: carbamylation of cysteines; variable modifications: oxidation (M), N-terminal acetylation and Gly to Asp; variable number of miscleavages: two. Data was searched against a human Fasta database, with a false discovery rate cutoff of 1% (forward and reverse) at both peptide and protein level. Protein abundance was calculated using an LFQ-algorithm. For Western blotting experiments the GST-RBD pulldowns were immunoblotted with an antibody against KRAS (BD Transduction Labs).

## Results

### Pipeline design and SNV analysis

**Fig 1:**
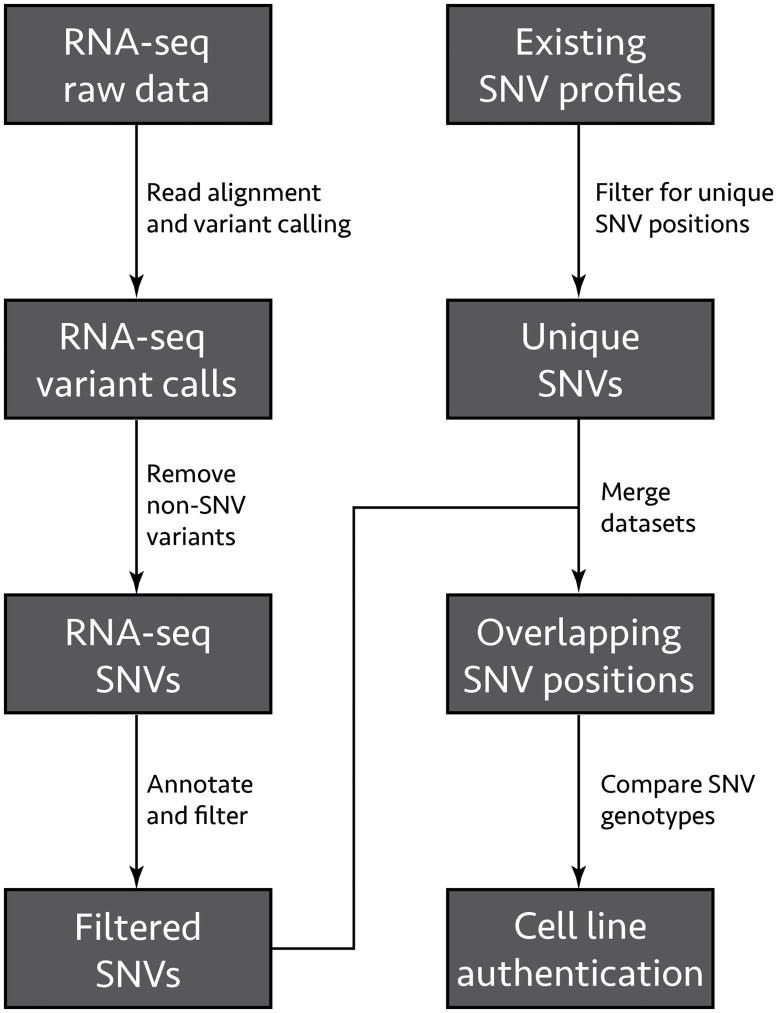
RNA-seq cell line authentication pipeline. Raw RNA-seq data is aligned to the human genome using STAR, followed by processing and variant calling steps using GATK tools. Non-SNV variants (insertions, deletions, *etc*.) are removed, and the resulting SNVs are annotated using *SnpEff* and *SnpSift*. SNVs passing the GATK variant filtration step and having a total allelic depth of at least 10 are compared to COSMIC SNV profiles filtered to include unique SNV positions, as well as a SNV genotyping panel.

Cell line contamination is a ubiquitous problem and can occur in several different ways. Mycoplasma contaminations has previously been investigated using RNA-seq data by identifying reads that map to the mycoplasma genome. [3] In addition, the use of RNA-seq data for SNV analysis has also been established by several studies. [14, 29–31] However, these studies have mainly been used for the analysis of transcriptome and genome variation. By analysing variants found in RNA-seq data, Strong *et al*. were able to interrogate the origin of several widely used lung cancer cell lines and identify HeLa contaminations. [4] Cirulli *et al*. has previously shown that variants from RNA-seq data cover 40% of those found from whole genome sequencing, and up to 81% when filtering for expressed genes. [29] The aim of this study is to evaluate the possibility of using RNA-seq SNV data for cell line authentication. To this end, we propose an authentication pipeline using existing RNA-seq variant calling procedures (which statistically evaluate possible SNVs and other variants in high-throughput sequencing data, such as insertions and deletions) in combination with annotation, filtering and comparisons with existing data from the COSMIC database (Fig 1). [15] COSMIC contains (among other cancer-related data) a manually curated database of between hundreds up to several thousands of unique SNVs for 1025 cell-line specific mutational profiles, which makes it particularly suited for comparisons with high-throughput sequencing data. Our authentication strategy comprises of comparisons between the genotypes of SNVs found in RNA-seq data with those found in the COSMIC database. This is analogous to the already well-established methods of STR profiling and SNV genotyping, but more fully uses the scope of the sequencing technologies available today, resulting in a more powerful authentication procedure.

The first step of the pipeline is the alignment of the raw sequencing reads using the *STAR* aligner. [20] The alignments are then used to find RNA-seq SNVs using variant calling procedures from the *Genome Analysis Toolkit* (GATK). [21] GATK has been shown to be accurate for both SNVs and indels, in addition to possessing explicit functionality for dealing with RNA-seq data. [32] The resulting SNVs are annotated using *SnpSift/SnpEff*, [22, 23] yielding information on SNV type and putative impact. SNV types are classified either as missense, nonsense or synonymous whereas the impacts are categorised either as HIGH, MODERATE, LOW or MODIFIER. SNVs classified as HIGH include variants that have a disruptive impact on protein sequence, probably causing protein truncation, loss of function or triggering nonsense-mediated decay. The MODERATE SNVs include non-disruptive variants that might change protein effectiveness whereas LOW impact SNVs are assumed to be mostly harmless or unlikely to change protein function. SNVs classified as MODIFIER are usually non-coding variants or variants affecting non-coding genes, where predictions are difficult or there is no evidence of impact.

To generate a SNV profile, the identified SNVs were filtered to only include those containing the highest impact variants for each transcript, as these are most likely to affect the biology of the cell lines. In order to assure a high statistical confidence of the generated SNV profiles, the variants failed by the GATK filters for strand bias and quality score are also removed, as are those with a total allelic depth less than 10. [30] The genotype of each unique position in the resulting SNV profile is then compared to the genotype of the same position in the COSMIC database, yielding information about cell line authenticity.

We have chosen to test the authentication pipeline on both novel and publicly available RNA-seq data from several cell lines, namely COLO205, DLD1, HCT15, HCT116, HKE3, HT29 and RKO. These are all widely used model systems for colorectal cancer and cover a broad range of cancer subtypes. COLO205 is related to several other COLO cell lines derived from the same adenocarcinoma patient. [33] The DLD1 and HCT15 cell lines are derived from the same patient with colon carcinoma by two independent researchers. They show similar genetic profiles and histological appearance but different karyotypic changes. [34–36] HCT116 is derived from colon carcinoma and contains a KRAS-G13D mutation, which is among the most ubiquitous mutations found in human cancers. [37] The HKE3 cell line is a KRAS-G13D knockout of HCT116, where the G13D allele has been altered by a disruption cassette. [38] The HCT116/HKE3 cell line pair thus constitutes a useful model of the KRAS-G13D mutation. HT29 is also derived from an adenocarcinoma. [39] RKO is a poorly differentiated colon carcinoma cell line. [40] The HCT116, HKE3 and RKO cell lines were sequenced in-house, whereas data for COLO205, DLD1, HCT15, HT29 and an additional dataset for HCT116 were retrieved from the Gene Expression Omnibus (GEO) database. [16]

An overview of the sequencing parameters can be seen in Table 1. The variant calling procedures yielded a total of 1 347 005 filtered SNVs for the eight cell lines. The highest number of SNVs (670 153) was achieved for the HKE3 cell line, which also has the highest number of reads (255 million read pairs) and the longest read length (125 bp), while the lowest number of SNVs (38 456 and 38 777, respectively) is for the HT29 and COLO205 cell lines (approximately 90 million 50 bp reads, each). Interestingly, the DLD1 cell line has 1947 SNVs per million reads (comparable to that of HCT116a, HKE3 and RKO) while having the fewest number of reads (37 million).

**Table 1:** Sequencing information and total number of SNVs found in the variant calling analysis for the different cell line experiments.

Cell line	Replicates	Read design	Total reads	Total SNVs	SNVs per 10^6^ reads
COLO205	3	50 x 1	89 964 067	38 777	431
DLD1	2	69 x 1	37 087 864	72 203	1947
HCT116a	3	100 x 2	98 756 403	177 948	1802
HCT116b	3	50 x 1	96 249 743	44 231	460
HCT15	3	50 x 1	97 492 596	55 195	566
HKE3	4	125 x 2	255 394 483	670 153	2624
HT29	3	50 x 1	87 846 283	38 456	438
RKO	3	100 x 2	103 479 468	250 042	2416

### SNV comparisons for cell line authentication

We compared RNA-seq SNV profiles from the eight cell lines with the COSMIC data on cell line-specific SNV profiles. While the COSMIC database lacks an entry for the DLD1 cell line, it is possible to validate its relationship with HCT15 by comparing the DLD1 data to the HCT15 COSMIC SNV profile. HKE3 similarly lacks a COSMIC profile, but its origin makes comparisons with the HCT116 profile possible. The data used in this study includes cell lines that have between 241 and 7649 unique SNVs in the COSMIC database, covering a large range of mutations. We define *concordance* as the proportion of RNA-seq SNVs matching those in the COSMIC SNV profiles (matching SNVs ÷ total SNVs). As can be seen in Table 2, all cell lines have a high concordance; the lowest concordance achieved for any of the eight cell lines is 96.2% (HKE3). This does not seem to correlate with the number of SNVs available in COSMIC; while the cell line with the highest concordance also has the most SNVs (HCT15), those with fewest SNVs both have concordance of at least 98.5% (COLO205 and HT29).

**Table 2:** Overview of the comparisons between RNA-seq SNVs, COSMIC SNVs and the Yu *et al*. genotyping panel.

Cell line	COSMIC SNVs	RNA-seq SNVs	Cov.	Conc.	HeLa SNVs	Yu SNPs
COLO205	241	68	28.2%	98.5%	0/1	2/2
DLD1	7649*	2239	29.3%	98.7%	0/1	2/2
HCT15	7649	3356	43.9%	99.3%	0/1	2/2
HCT116a	2428	1122	46.2%	97.5%	0/1	2/2
HCT116b	2428	1003	41.3%	98.3%	0/2	2/2
HKE3	2428*	1379	56.8%	96.2%	0/2	7/7
HT29	462	145	31.4%	98.6%	0/0	2/2
RKO	2676	1112	41.6%	96.5%	0/2	2/2

Coverage (cov.) is defined as the number of COSMIC SNVs that are found in the RNA-seq data, and concordance (conc.) is defined as the proportion of RNA-seq SNVs that match the genotype in COSMIC.

*DLD1 and HKE3 based on COSMIC HCT15 and HCT116 profiles, respectively.

As the COSMIC database also contains data on non-expressed variants, the RNA-seq data will not cover all of the SNVs listed. We define *coverage* as the proportion of COSMIC SNVs that are found by the RNA-seq analysis (filtered RNA-seq SNVs ÷ unique COSMIC SNVs). The average coverage of the analysed cell lines is 39.8% (Table 2). The cell line with the highest sequencing depth (HKE3) also has the highest coverage (56.8%), but even DLD1 with its relatively few 37 million reads achieves a coverage of 29.3%, similar to that of COLO205 and HT29.

**Fig 2:**
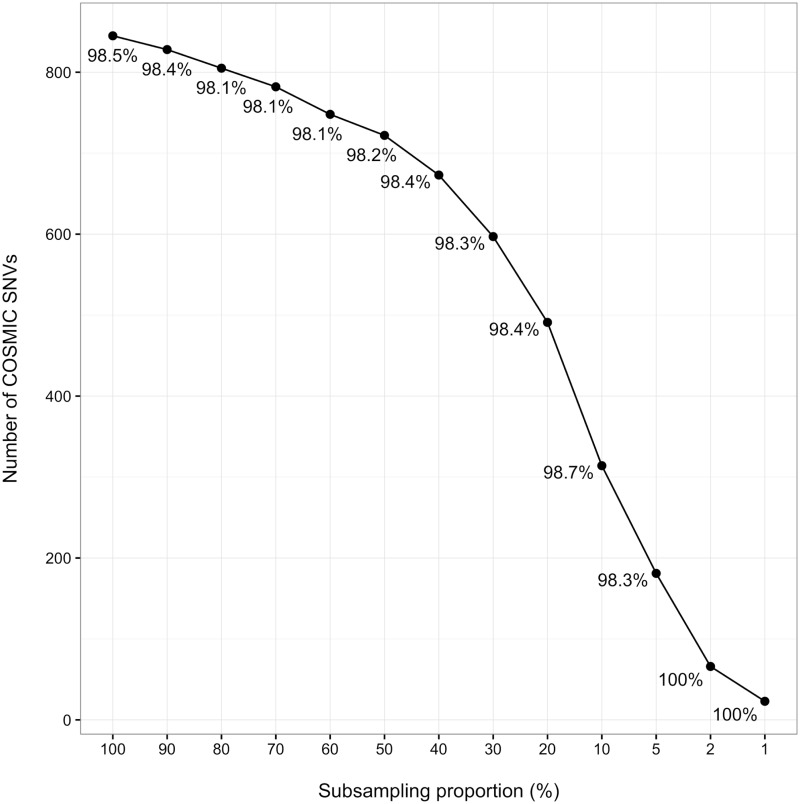
Subsampling of RNA-seq data. The effect of subsampling on the number and concordance of the RNA-seq SNVs. Here, decreasing subsets of a single replicate of HCT116a (corresponding to a total of 32 million read pairs) is shown. The concordance of each subset is annotated next to the corresponding data point. For data on additional cell lines, see S1 Fig.

In order to further test the effect of sequencing depth on the results, we ran the pipeline through subset of a single replicate of the HCT116a cell line. As can be seen in Fig 2, the number of SNVs are sufficient for authentication even for smaller subsets; the 2% subset corresponds to approximately 640 000 read pairs and yields a 100% concordance for its 66 SNVs. The subsets from five of the other cell lines yield similar results (S1 Fig), demonstrating that the pipeline is robust to low sequencing depths.

Contaminations of HeLa cells is one of the most common problems related to cell line authenticity, previously shown to account for 29% of all contaminations in human cell lines. [11] By comparing the RNA-seq SNVs with the HeLa COSMIC SNV profile rather than that of the correct profile, it is also possible to check for HeLa contaminations. As can be seen in Table 2 between zero and up to two HeLa SNVs are found in the investigated cell lines, all with a 0% concordance. Regardless of the fact that the COSMIC HeLa profile contains only 379 unique SNVs, this data clearly show that these cell lines are free from HeLa contamination.

As a control, the RNA-seq SNV data for the various cell lines were compared to incorrect COSMIC SNV profiles (S2 Fig). As some of the comparisons have a 100% concordance but a very low coverage, this data shows the importance of having both high coverage and high concordance to evaluate authenticity when comparing with COSMIC SNV profiles. In order to confirm the authenticity of the analysed cell lines with another source, the SNVs were compared to a recent SNP genotyping panel for cell line authentication published by Yu and colleagues. [9] This panel covers 48 loci selected based on minor allele frequency and availability on commercial genotyping platforms. While the concordance of this comparison was 100% for all eight cell lines, they cover few of the SNPs available in the panel (Table 2). This data illustrates the power of using transcriptome-based SNVs in combination with the COSMIC profiles, which captures the global nature of RNA-seq data more comprehensively.

**Fig 3:**
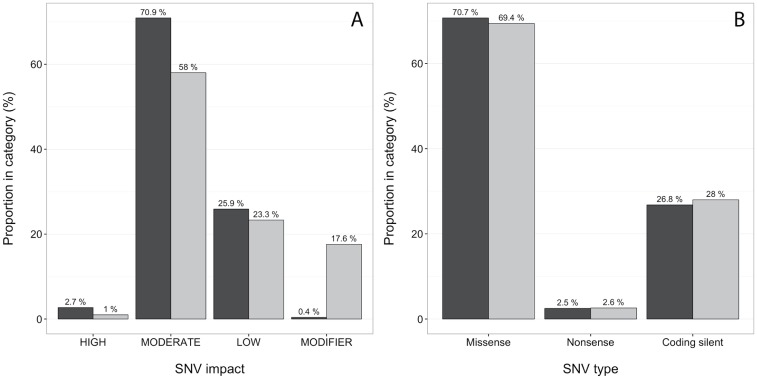
SNV characterisations. Characterisation of the RNA-seq matching (black) and mismatched (grey) SNVs for all cell lines compared to COSMIC profiles. Counts for each category is shown above each bar. **(A)** Proportions of SNV impact. **(B)** Proportions of mutation type.

In order to characterise and evaluate the differences between the RNA-seq SNVs and COSMIC profiles, we investigated the relative abundances of different annotation categories for matching and mismatched SNVs. As can be seen in Fig 3A, there are more than twice as many matching HIGH impact SNVs as for the mismatched ones (2.7% and 1.0%, respectively), and more MODERATE SNVs as well (for characterisation of individual cell lines, see S3 Fig). The most pronounced difference for SNV impact is for the MODIFIER category, where there are 0.4% matches and 17.6% mismatches. There is no difference between matching and mismatched SNVs for any SNV type category (Fig 3B). Absolute numbers for the relative comparisons presented in Fig 3 can be found in Table 3.

**Table 3:** Absolute numbers of SNV characterisation categories, for a total of 10231 matching and 193 mismatching SNVs.

Category	SNV Matches	SNV Mismatches
HIGH	280	2
MODERATE	7254	112
LOW	2651	45
MODIFIER	46	34
Missense	7238	134
Nonsense	255	5
Coding silent	2738	54

**Fig 4:**
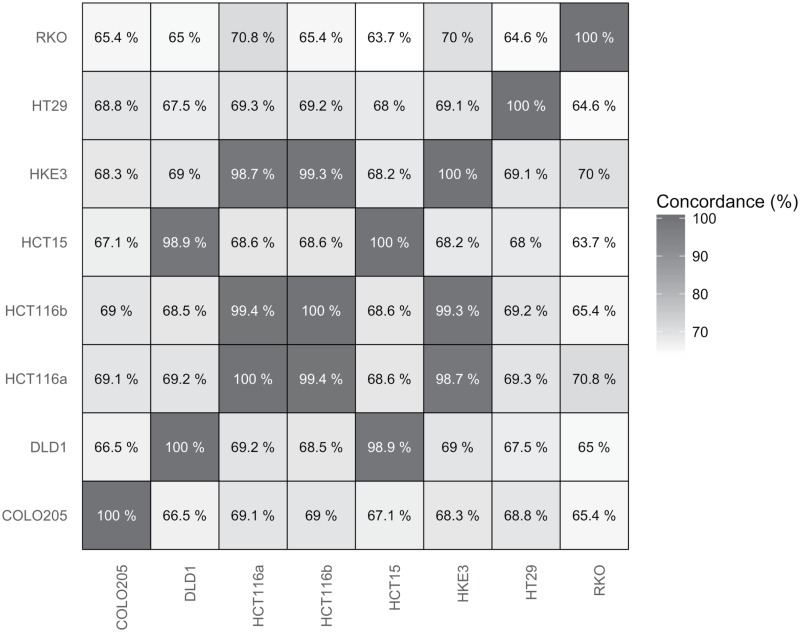
Transcriptome-wide comparisons. Comparisons of transcriptome-scale SNV profiles for every cell line investigated, with a colour gradient for increasing concordance. The average concordance of non-related cell lines is 67.9%.

A large proportion of the RNA-seq variant data corresponds to SNVs not present in the COSMIC database. Approximately 1 34 7005 SNVs were found for all eight cell lines in total, while only 10 500 of these were found in COSMIC. We thus sought to evaluate the possibility of authenticating cell lines by comparing their transcriptome-scale SNV profiles. Fig 4 show comparisons between all eight cell lines in this study, where every overlapping SNV was analysed for matching genotypes. Comparisons of highly related cell lines leads to very high concordance (such as for HCT116 and HKE3, 98.7%), while comparisons of the same data lead to a 100% concordance, as expected. The average concordance for comparisons of non-related cell lines is 67.9%.

As the cell lines DLD1 and HCT15 are derived from the same patient and have a similar genetic profile, is it possible to check the validity of the method by comparing them to each other. As DLD1 show a 98.7% concordance with the HCT15 COSMIC profile, it is clear that the pipeline is working as expected and can confirm already established conclusions. HKE3 also shows a 96.2% concordance with its parental cell line, HCT116. Surprisingly, the KRAS-G13D mutation is not only found in HCT116 COSMIC comparison as expected, but also in HKE3. This suggests that the KRAS-G13D allele is not fully abolished, leading to expression of this mutant on the RNA level.

### Expression of KRAS variants in HKE3

To further clarify the KRAS mutations carried by the HKE3 cell line we investigated it in detail on sequence level. HKE3 is a HCT116 disruption mutant generated by homologous recombination aimed at deletion of the KRAS-G13D mutant and insertion of a non-transcribed KRAS-G12C mutant. By matching the aligned reads generated by the alignment step to either G12C, G13D or wild type, we can get an indication of the KRAS read distribution in the samples, as shown in Table 4. While the distribution is slightly skewed in favour of wild type (wt) reads for HCT116, the ratio is approximately 1:1 (wt:G13D), as expected. The distribution for HKE3 is less obvious, and reads corresponding to wt, G12C and G13D are detected with ratios close to 1:1:1. This indicates that the disruption cassette may have been incorporated in a position that still allows expression of the G13D mutant, rather than in the KRAS gene as was intended. Alternatively, a *de novo* mutation in KRAS could have occurred during the culturing of the HKE3 cells. However, the continued presence of wt KRAS means that such a *de novo* mutation would have required gene duplication of the wt KRAS allele first. As expected, no reads contained both the G12C and the G13D mutations (data not shown). These results were validated by PCR amplification and Sanger sequencing of KRAS exon 2 using RNA from both HCT116 and HKE3 (S4 Fig). Two antibiotic resistance genes Neomycin (Neo) and Thymidine Kinase (TK), which are part of the disruption cassette designed to remove the KRAS-G13D mutation, have successfully been incorporated into the genome and are being expressed on the RNA level in HKE3, as expected (S5 Fig). We also replicated the validation experiment as performed by Shirasawa *et al*. (S6 Fig), yielding similar results. As no KRAS-G12C mutation could been seen in the first Sanger sequencing reaction, a second reaction with separate primers for the wild type KRAS and TK show that this cassette-originating mutation in indeed present on the RNA level (S7 Fig), corroborating the RNA-seq analysis. All PCR experiments have been performed by two separate labs.

**Table 4:** Aligned KRAS read distribution for HCT116 and HKE3.

Replicate	12wt / 13wt	12wt / 13mut	12mut / 13wt	Total
HCT116 a	40	40	0	80
HCT116 b	36	34	0	70
HCT116 c	60	36	0	96
HCT116 mean	55.3%	44.7%	0.0%	
HKE3 a	82	46	35	163
HKE3 b	55	46	30	131
HKE3 c	32	62	44	138
HKE3 d	31	48	38	117
HKE3 mean	36.4%	36.8%	26.8%	

The proportion of each read combination for each cell line is given in the last three columns. The approximate ratio of 1:1:0 (wt:G13D:G12C) for HCT116 is expected, but the ratio is closer to 1:1:1 for HKE3.

**Fig 5:**
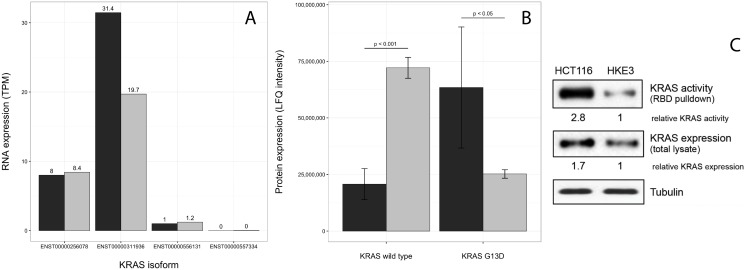
KRAS expression levels. **(A)** RNA expression levels of the four KRAS isoforms in HCT116 (black) and HKE3 (grey). While KRAS is less expressed in HKE3 than in HCT116, the isoform expression analysis indicates that it is only a single KRAS isoform with an expression difference, while the other three remain unchanged. **(B)** Protein levels from RBD pulldown and MS quantification (error bars are based on the standard error of the mean), showing that the G13D mutation is expressed on the protein level in both HCT116 and, at a lower level, HKE3. **(C)** KRAS expression and activity in HCT116 and HKE3 cells. Active KRAS was pulled down from lysates of serum starved cells with GST-RBD beads. Aliquots of the lysates were blotted for total KRAS and tubulin as loading control. Western blots were quantified using *Image J*. Relative KRAS activity was normalised to KRAS expression levels, which were normalised to tubulin expression levels.

Furthermore, the KRAS-G13D mutant seems to be less expressed in HKE3 compared to HCT116. In order to quantify the levels of KRAS transcripts in HCT116 and HKE3, differential expression analysis for the two cell lines was performed. A fold change of 1.89 (FDR = 0.006) shows that the KRAS gene is less expressed in HKE3 than in HCT116. An isoform expression analysis using Kallisto [26] show that it is a single KRAS isoform (ENST00000311936) out of four known that differ in expression level between these cell lines (Fig 5A).

As the fold change of KRAS-G13D between HCT116 and HKE3 is below the common threshold of 2 for biological significance, we sought to validate this down-regulation on the protein level. Quantification of KRAS-G13D protein levels was performed using a Ras Binding Domain (RBD) pulldown assay followed by Mass Spectrometry (MS), which show a clear difference in the levels of G13D expression between HCT116 and HKE3 (Fig 5B). Western blots show a 1.7 relative fold increase of KRAS expression in HCT116, similar to the differential expression analysis, and a full 2.8 fold increase of KRAS activity (Fig 5C). This data indicates that HKE3 is a dosage effect mutant in regards to KRAS-G13D, rather than a full knockout. While this may not be the original intention of establishing HKE3, analysing the cell line pair can still provide valuable information as to how small differences in KRAS-G13D expression may affect the cell.

## Discussion

Cell line authentication is an important and non-trivial problem in biological research. While mycoplasma infections and inter-species contaminations do happen and can affect the outcome and interpretation of experimental results, the more common problem is that of cross-contamination with other human cell lines. There are numerous problems associated not only with the technical aspects of cell line authentication, but also in defining standards and best practice procedures, implementation by researchers world-wide as well as curation of data repositories storing experimental results performed with non-authenticated cells. [6] An increasing number of journals are requesting confirmatory tests for cell line authenticity. Methods for finding mycoplasma infections [3] and cross-species contaminations [4] using RNA-seq data has already been established, but novel technologies and analysis methods for cell line authentication are still needed, in addition to the ones already established. [12] Comparisons of RNA-seq SNVs with existing SNV profiles can be one such novel technology. To explore this possibility, we have created a pipeline for cell line authentication that uses the power of transcriptome-wide sequence information from RNA-seq experiments. The method is based on generating a SNV profile based on the RNA-seq data and comparing it with already published SNV profiles in the COSMIC database. This strategy opens the possibility to assess the authenticity of cell lines in already reported experimental results and to revisit cell authenticity in repositories of RNA-seq data. As many more RNA-seq SNVs than those present in the COSMIC profiles were found, it is also possible to create new and more comprehensive SNV profiles from the RNA-seq data.

We have shown that our pipeline is robust to different sequencing parameters, including single or paired-end reads, varying read lengths as well as for different sequencing depths. The lowest concordance for any of the eight cell lines examined herein was 96.2%, while going as high as 99.3%. Yu *et al*. proposed a threshold of 90% concordance for their 48-locus SNP array with a coverage of at least 85%. Even the cell line with the fewest COSMIC SNVs in our data (COLO205, 68 SNVs) pass this threshold, demonstrating the strength and validity of our strategy. The average number of SNVs for the cell lines presented here is 1303, while the average number of unique SNVs in the COSMIC database as a whole is 666. These numbers attest to the power of using high-through sequencing data for cell line authentication. Cirulli *et al*. found that approximately 40% of genomic SNVs were captured by RNA-seq, making our average coverage of 39.8% in line with previous data. [29] These results show that a full coverage of genomic variants is not needed and that RNA-seq data is more than sufficient for cell line authentication.

As sequencing depth increases, so does the number of SNVs, but even experiments with relatively few reads can be used successfully for cell line authentication with our pipeline. The cell line with the fewest reads (DLD1, 37 million) has approximately 72 000 filtered SNVs in total and a coverage of 29.3%, similar to those cell lines with around 90 million reads. The majority of the experiments with a shorter read length (COLO205, HT29) also has fewer SNVs in total (approximately 38 000 each) and a lower coverage compared to those with longer read lengths. The exception is HCT15, which has a coverage of 43.9%. This is most likely due to the large number of COSMIC SNVs available for HCT15 (7649 unique SNVs), compared to those available for COLO205 and HT29 (241 and 462 unique SNVs, respectively). Another study analysed 81 million reads, which resulted in approximately 40 000 variants that passed their quality controls, leading to 501 SNVs per million reads. [29] This is comparable to the cell lines investigated in this study.

The analysis of the data subsets show that the coverage decreases with lowered sequencing depth, while maintaining a high concordance. This shows that the coverage is affected by both the sequencing parameters and the number of variants in the COSMIC database. The robustness of the pipeline is further demonstrated by the fact that this variable coverage is not concomitant with lowered concordance. However, if there are too few SNVs found the authenticity will be less clear-cut, regardless of concordance. While the characterisation of the SNVs show that the impacts are relatively similar, there is a higher proportion of SNVs in the MODIFIER category for the mismatched SNVs than for the matching ones. Both the HIGH and MODERATE categories, however, had a smaller proportion in the mismatched SNVs.

One of the strengths of using RNA-seq data for cell line authentication compared to existing methods is that the sequence information itself allows for more in-depth analysis. The results from our pipeline indicated that one of the mutations in HKE3 didn’t correspond to what was known about the cell line, warranting a more thorough investigation. HKE3 was derived from HCT116 using a disruption cassette, which was intended to replace the original KRAS-G13D mutation in HCT116 with a non-expressed KRAS-G12C mutation. The results show that while the cassette has successfully been incorporated in the genome of HKE3, it does not entirely disrupt the expression of either KRAS allele. Data from RNA-seq, mass spectrometry as well as PCRs and Sanger sequencing on both the DNA and RNA level from two separate labs show that the HKE3 cells investigated here harbour a KRAS-G13D dosage effect, rather than a full knockout. The deviation from a 1:0:1 (wt:G13D:G12C) ratio of the distribution of aligned reads to 1:1:1 indicates that the cassette may have been incorporated elsewhere on the genome. As HCT116 has a higher rate of proliferation than HKE3, it is unlikely that the reported results are due to a HCT116 contamination. In the case of a contamination, HCT116 would overtake the HKE3 population, and no KRAS-G12C mutation would be seen. Another possible explanation is that a *de novo* mutation may have been introduced during culturing of HKE3, or that a chromosomal re-arrangement has taken place. However, defining the exact mechanism by which HKE3 expresses the KRAS-G13D mutation is beyond the scope of this study. While this was not the intended effect of the disruption cassette, the resulting cell line is still of great interest, as it can provide novel insights into the function of KRAS-G13D dosage and its effect on cellular signalling.

The cell lines DLD1 and HCT15 have previously been shown to be related. According to the SNP profiling from Yu and colleagues, these are classified as synonymous cell lines, *i*.*e*. they are derived from the same patient. One out of 48 loci in the SNP panel differ between the cell lines: the mutation *rs2355988*, for which the genotype of HCT15 is G/C, while that of DLD1 is C/C. While this particular mutation cannot be seen in the RNA-seq data, the concordance of DLD1 with the COSMIC HCT15 profile is 98.7%. The existing conclusion that DLD1 and HCT15 are synonymous cell lines is thus confirmed and demonstrates how established results can be re-evaluated by our authentication pipeline. Contaminations of HeLa cells were also ruled out for all of the studied cell lines. While outside the scope of this article, the general applicability of the method makes it possible to check for contaminations of any other cell line for which there exists a SNV profile, in both current and future databases.

As RNA-seq SNV data contains both global and more in-depth data than previously established authentication methods such as STR profiling and SNV genotyping panels, our pipeline could potentially replace said methods when RNA-seq data is available. Its strengths lie in the fact that many more variants are discovered and thus give a more complete picture of the cell lines analysed, even for those cases where relatively few variants are listed in the COSMIC database. While such a replacement has theoretical merits, the cost of RNA-seq is not yet low enough to warrant spending the money purely for cell line authentication. However, studies aiming to use RNA-seq data for gene expression analyses can benefit from performing cell line authentication using our pipeline on the same data. There are great opportunities to retroactively authenticate and validate the numerous already published RNA-seq datasets in the literature using the presented methodology. There also lies considerable potential in comparing transcriptome-scale SNV profiles, especially for those cell lines where no COSMIC profile exist. Creating such profiles would allow for authentication of any cell line for which RNA-seq is (or becomes) available. Both existing and future RNA-seq data from cell line experiments could be used to create such profiles using our pipeline, fully utilising the strength and scope of the sequencing technology.

## Conclusion

We have created a pipeline for cell line authentication using RNA-seq data, shown its robustness to several sequencing parameters, used it to validate already existing conclusions regarding cell line authenticity as well as shown how such data can be utilised for detailed analysis of cell lines with known mutations. Our pipeline was used to confirm that the cell lines DLD1 and HCT15 are synonymous, and we have also shown that the HKE3 cell line, derived from HCT116, harbours a KRAS-G13D dosage effect rather than a full knockout. This information can be used to conduct new experiments to gain insight into the effect of KRAS-G13D on the cell and its affected pathways. The inherent power of high-throughput sequencing technologies gives our pipeline the potential to replace already existing authentication methods, but may also be used to develop novel transcriptome-scale SNV profiles for authentication based on already existing cell line RNA-seq data.

## Supporting information

S1 Fig Subsampling of individual cell lines. The effect of subsampling single replicates (corresponding to approximately 30 million reads) to a lower read depth on cell line authentication statistics; COLO205 (blue), HCT116a (orange), HCT116b (light blue), HCT15 (yellow), HT29 (green), RKO (red). The concordance for all subsampling proportions for all cell lines remains above 90% except in two cases: the 2% and 1% subsamples of COLO205, where the number of filtered SNVs were zero. (**A**) Number of SNVs as dependent on subsampling proportion; (**B**) concordance of filtered SNVs as dependent on subsampling proportion.
(EPS)

S2 Fig COSMIC coverage and concordance heatmap. Heatmap showing the coverage of RNA-seq SNVs compared to those in COSMIC profiles (colour gradient and second percentage in boxes) and the concordance of these SNVs (first percentage in boxes).
(EPS)

S3 Fig Impact characterisation of individual cell lines. Characterisation of the RNA-seq matching (black) and mismatched (grey) SNV impacts for individual cell lines compared to COSMIC profiles. Counts for each category is shown above each bar.
(EPS)

S4 Fig PCR of KRAS exon 1. **(A)** PCR amplification and Sanger sequencing of KRAS exon 2 (KRASF1 + KRASR2) of RNA from **(1)** HCT116 and **(2)** HKE3. Sanger sequencing of KRAS-G13D in **(B)** HCT116 and **(C)** HKE3.
(EPS)

S5 Fig PCR of the disruption cassette. PCR amplification of the disruption cassette in HCT116 and HKE3 using NeoF1, NeoR1, TkF1 and TkR1 primers. **(1–4)**: HCT116 Neo, HCT116 TK, HKE3 Neo, HKE3 TK (DNA); **(5–8)**: same as 1–4, but with cDNA. Ladder from below: 250, 500, 750 and 1000 bp.
(EPS)

S6 Fig PCR of the disruption cassette with original primers. PCR amplification of the non-disrupted (170bp, red arrow) and disrupted (4.2kb, blue arrow) KRAS locus cassette in **(1)** HCT116 and **(2)** HKE3 using PKF1 and PKR1 primers according to Shirasawa *et al*. [38].
(EPS)

S7 Fig PCR of WT/TK-specific KRAS regions. PCR amplification of either **(1)** wild type (KRASFwt + KRASR3) or **(2)** TK (KRASFTk + KRASR3) transcripts in HKE3 RNA. Ladder from below: 250, 500, 750 and 1000 bp.
(EPS)

S1 File S1_File.txt. List of primer sequences used in the study.
(TXT)

S2 File Cell authentication.Rmd. The R code used to create the figures and tables in the study.
(PDF)
