# Supplementary material for: A novel RNA sequencing data analysis method for cell line authentication
Source: PLoS One. 2017 Feb 13;12(2):e0171435. doi: 10.1371/journal.pone.0171435 (PMC5305277; doi:10.1371/journal.pone.0171435)
Supplement: S2 File — The R code used to create the figures and tables in the study. (PDF) [file pone.0171435.s009.pdf]

# RNA-seq data analysis for cancer cell line authentication

This R Markdown document describes the analyses performed in order to reach the data, figures and tables included in the article. Annotated variant calling data for the COLO205 cell line is included and used to show the general workflow of the authentication pipeline, as well as files of the collected authentication data for all eight cell lines used in this study.

## Load and pre-process variant calling data

The file *variant.calls.colo205.txt* is included in the R Markdown directory.

```
data = read.table('variant.calls.colo205.txt', sep='\t', quote=NULL, comment='',
                  stringsAsFactor=FALSE)
names(data) = c('chr', 'pos', 'rsID', 'REF', 'ALT', 'gene', 'ENSTID', 'ENSGID',
             'impact', 'effect', 'feature.type', 'transcript.biotype',
             'sample_1', 'sample_2','DP', 'filter', 'AD', 'nucleotide',
             'amino.acid', 'warnings')
data$ALT = gsub('\\[|\\]', '', data$ALT)

# Remove duplicated positions and indels; add sample name.
data = data[!duplicated(data[, c('chr', 'pos', 'ENSGID')]), ]
data = data[(nchar(data$sample_1) == 1 & nchar(data$sample_2) == 1), ]
data$sample = 'COLO205'

# Convert to GRanges object for quick and efficient comparison with COSMIC data.
suppressPackageStartupMessages(library("GenomicRanges"))
data.gr =
  makeGRangesFromDataFrame(data, keep.extra.columns=TRUE,
                           ignore.strand=TRUE, seqinfo=NULL,
                           seqnames.field='chr', start.field="pos",
                           end.field="pos", starts.in.df.are.0based=FALSE)

# Keep only chromosomes 1 through 22, X and Y.
seqlevels(data.gr) = gsub("chr", "", seqlevels(data.gr))
data.gr = keepSeqlevels(data.gr, c(as.character(1:22), 'X', 'Y'))
data.gr
```

```
## GRanges object with 87441 ranges and 19 metadata columns:
##          seqnames                 ranges strand   |        rsID
##             <Rle>              <IRanges>  <Rle>   | <character>
##        1        1         [14930, 14930]      *   |  rs75454623
##        3        1         [14930, 14930]      *   |  rs75454623
##       10        1         [14933, 14933]      *   | rs199856693
##       12        1         [14933, 14933]      *   | rs199856693
##       19        1         [20129, 20129]      *   | rs202081272
##      ...      ...                    ...    ... ...         ...
##   370640        X [154350245, 154350245]      *   |        None
##   370641        X [154350245, 154350245]      *   |        None
##   370645        Y [ 21152851,  21152851]      *   |        None
##   370647        Y [ 21152851,  21152851]      *   |        None
##   370648        Y [ 21152851,  21152851]      *   |        None
```

```
##                  REF         ALT        gene          ENSTID
##          <character> <character> <character>     <character>
##       1            A           G     DDX11L1 ENST00000515242
##       3            A           G      WASH7P ENST00000538476
##      10            G           A     DDX11L1 ENST00000518655
##      12            G           A      WASH7P ENST00000541675
##      19            C           T      WASH7P ENST00000438504
##     ...          ...         ...         ...             ...
##  370640            G        None       BRCC3 ENST00000340647
##  370641            G        None       MTCP1 ENST00000476116
##  370645            T        None      TTTY14 ENST00000452584
##  370647            T        None      CD24P4 ENST00000382840
##  370648            T        None    ZNF839P1 ENST00000403487
##                   ENSGID      impact                 effect feature.type
##              <character> <character>            <character>  <character>
##       1 ENSG00000223972    MODIFIER downstream_gene_variant   transcript
##       3 ENSG00000227232    MODIFIER          intron_variant   transcript
##      10 ENSG00000223972    MODIFIER downstream_gene_variant   transcript
##      12 ENSG00000227232    MODIFIER          intron_variant   transcript
##      19 ENSG00000227232    MODIFIER          intron_variant   transcript
##     ...             ...         ...                     ...          ...
##  370640 ENSG00000185515    MODIFIER downstream_gene_variant   transcript
##  370641 ENSG00000214827    MODIFIER          intron_variant   transcript
##  370645 ENSG00000176728    MODIFIER          intron_variant   transcript
##  370647 ENSG00000185275    MODIFIER downstream_gene_variant   transcript
##  370648 ENSG00000217896    MODIFIER downstream_gene_variant   transcript
##                        transcript.biotype    sample_1    sample_2
##                               <character> <character> <character>
##       1 transcribed_unprocessed_pseudogene           A           G
##       3             unprocessed_pseudogene           A           G
##      10 transcribed_unprocessed_pseudogene           G           A
##      12             unprocessed_pseudogene           G           A
##      19             unprocessed_pseudogene           C           T
##     ...                                ...         ...         ...
##  370640                     protein_coding           G           G
##  370641               processed_transcript           G           G
##  370645                            lincRNA           T           T
##  370647               processed_pseudogene           T           T
##  370648               processed_pseudogene           T           T
##                 DP      filter          AD    nucleotide  amino.acid
##          <integer> <character> <character>   <character> <character>
##       1        13        None      [6, 7]    n.*1653A>G
##       3        13        None      [6, 7]  n.1491+70T>C
##      10        13        None      [9, 4]    n.*1483G>A
##      12        13        None      [9, 4]   n.949+37C>T
##      19        38        None    [28, 10] n.205-1750G>A
##     ...       ...         ...         ...           ...         ...
##  370640        59        None          59            c.
##  370641        59        None          59            n.
##  370645       140        None         140            n.
##  370647       140        None         140            n.
##  370648       140        None         140            n.
##             warnings      sample
##          <character> <character>
```

```
##         1                 COLO205
##         3                 COLO205
##        10                 COLO205
##        12                 COLO205
##        19                 COLO205
##       ...          ...        ...
##    370640                 COLO205
##    370641                 COLO205
##    370645                 COLO205
##    370647                 COLO205
##    370648                 COLO205
##   -------
##   seqinfo: 24 sequences from an unspecified genome; no seqlengths
```

### Load COSMIC data

The file *CosmicCLP_MutantExport.txt* (GRCh37 assembly) is included in the R Markdown directory, but can also be downloaded at the COSMIC website. (Forbes et al. 2015)

```
cosmic.all = read.table('CosmicCLP_MutantExport.txt', header=TRUE, sep='\t',
                        quote=NULL, comment='', stringsAsFactors=FALSE)
cosmic.all = cosmic.all[c('Gene.name', 'Accession.Number', 'Sample.name',
  'Mutation.ID', 'Mutation.CDS', 'Mutation.AA', 'Mutation.Description',
  'Mutation.zygosity', 'Mutation.genome.position', 'strand',
  'Mutation.somatic.status', 'Mutation.verification.status')]
names(cosmic.all) = tolower(names(cosmic.all))

# Subset for COLO205 data.
cosmic.all$sample.name = tolower(gsub('[-_. ]', '', cosmic.all$sample.name))
cosmic = cosmic.all[grep('colo205', cosmic.all$sample.name), ]

# Remove duplicated positions and indels; store number of unique SNPs.
cosmic$gene.name = gsub('_ENST\\d+', '', cosmic$gene.name)
cosmic = cosmic[!duplicated(cosmic[, c('gene.name',
                                       'mutation.genome.position')]), ]
cosmic = cosmic[grep('Substitution', cosmic$mutation.description), ]
n.unique.snps = dim(cosmic)[1]

# Separate chromosomes and positions.
positions = data.frame(do.call(rbind, strsplit(as.vector(
  cosmic$mutation.genome.position), split = ":|-")))
names(positions) = c('chr', 'start', 'end')
cosmic = cbind(cosmic, positions)
cosmic$start = as.numeric(as.character(cosmic$start))
cosmic$end = as.numeric(as.character(cosmic$end))

# Get reference and alternative nucleotides.
mut = as.character(cosmic$mutation.cds)
cosmic$REF.cosmic = substr(cosmic$mutation.cds, nchar(mut)-2, nchar(mut)-2)
cosmic$ALT.cosmic = substr(cosmic$mutation.cds, nchar(mut), nchar(mut))

# Complement the reverse strand mutations for comparison with all-forward VCF.
idx = row.names(subset(cosmic, strand=='-'))
```

```r
cosmic[idx, 'REF.cosmic'] = chartr('ATCG','TAGC', cosmic[idx, 'REF.cosmic'])
cosmic[idx, 'ALT.cosmic'] = chartr('ATCG','TAGC', cosmic[idx, 'ALT.cosmic'])
cosmic$strand = NULL

# Get the COSMIC alleles.
cosmic$cosmic_1 = cosmic$REF.cosmic
cosmic[cosmic$mutation.zygosity == 'hom', 'cosmic_1'] =
  cosmic[cosmic$mutation.zygosity == 'hom', 'ALT.cosmic']
cosmic$cosmic_2 = cosmic$ALT.cosmic

# Convert to GRanges object and remove unwanted chromosomes.
cosmic.gr =
  makeGRangesFromDataFrame(cosmic, keep.extra.columns=TRUE,
                         ignore.strand=TRUE, seqinfo=NULL,
                         seqnames.field='chr', start.field="start",
                         end.field="end", starts.in.df.are.0based=FALSE)
seqlevels(cosmic.gr, force=TRUE) = c(as.character(1:22), 'X', 'Y')
cosmic.gr
```

```
## GRanges object with 235 ranges and 15 metadata columns:
##           seqnames                 ranges strand   |   gene.name
##              <Rle>              <IRanges>  <Rle>   | <character>
##      2523        7 [140453136, 140453136]      *   |        BRAF
##      5776        9 [119976858, 119976858]      *   |       ASTN2
##     16739       10 [ 50369665,  50369665]      *   |   C10orf128
##     18114        9 [ 90263726,  90263726]      *   |       DAPK1
##     25195        5 [140256399, 140256399]      *   |     PCDHA12
##       ...      ...                    ...    ... ...         ...
##    973795       11   [ 4936069,  4936069]      *   |      OR51G2
##    978788       11   [57268637, 57268637]      *   |     SLC43A1
##    980288       11   [56185040, 56185040]      *   |       OR5R1
##   1009268       16   [21893168, 21893168]      *   |   LOC440345
##   1019245       19   [14181774, 14181774]      *   |     EEF1DP1
##           accession.number sample.name mutation.id mutation.cds
##                <character> <character> <character>  <character>
##      2523  ENST00000288602     colo205      COSM476    c.1799T>A
##      5776  ENST00000361209     colo205    COSM69737      c.794G>A
##     16739  ENST00000474718     colo205   COSM296662      c.272G>A
##     18114  ENST00000358077     colo205   COSM319793    c.1360C>T
##     25195  ENST00000398631     colo205   COSM590012    c.1342G>A
##       ...              ...         ...         ...          ...
##    973795  ENST00000322013     colo205 COSM4192729     c.825C>T
##    978788  ENST00000278426     colo205 COSM4197407     c.320G>A
##    980288  ENST00000312253     colo205 COSM4196194     c.669C>T
##   1009268      XM_496125.1     colo205 COSM4257525    c.3584A>T
##   1019245  ENST00000344943     colo205 COSM4278511     c.565C>G
##           mutation.aa        mutation.description mutation.zygosity
##           <character>                 <character>       <character>
##      2523     p.V600E      Substitution - Missense               het
##      5776     p.R265Q      Substitution - Missense               het
##     16739      p.R91Q      Substitution - Missense               het
##     18114     p.R454C      Substitution - Missense               het
##     25195     p.V448M      Substitution - Missense               het
```

```
##       ...         ...                        ...              ...
##    973795     p.H275H Substitution - coding silent              het
##    978788     p.R107Q      Substitution - Missense              het
##    980288     p.A223A Substitution - coding silent              het
##   1009268    p.K1195I      Substitution - Missense              het
##   1019245     p.P189A      Substitution - Missense              het
##           mutation.genome.position
##                        <character>
##      2523    7:140453136-140453136
##      5776    9:119976858-119976858
##     16739     10:50369665-50369665
##     18114      9:90263726-90263726
##     25195    5:140256399-140256399
##       ...                      ...
##    973795       11:4936069-4936069
##    978788     11:57268637-57268637
##    980288     11:56185040-56185040
##   1009268     16:21893168-21893168
##   1019245     19:14181774-14181774
##                              mutation.somatic.status
##                                          <character>
##      2523 Reported in another cancer sample as somatic
##      5776 Reported in another cancer sample as somatic
##     16739 Reported in another cancer sample as somatic
##     18114 Reported in another cancer sample as somatic
##     25195 Reported in another cancer sample as somatic
##       ...                                          ...
##    973795                    Variant of unknown origin
##    978788                    Variant of unknown origin
##    980288                    Variant of unknown origin
##   1009268                    Variant of unknown origin
##   1019245                    Variant of unknown origin
##           mutation.verification.status  REF.cosmic  ALT.cosmic    cosmic_1
##                            <character> <character> <character> <character>
##      2523                     Verified           A           T           A
##      5776                     Verified           C           T           C
##     16739                     Verified           C           T           C
##     18114                   Unverified           C           T           C
##     25195                   Unverified           G           A           G
##       ...                          ...         ...         ...         ...
##    973795                   Unverified           G           A           G
##    978788                   Unverified           C           T           C
##    980288                   Unverified           G           A           G
##   1009268                   Unverified           T           A           T
##   1019245                   Unverified           C           G           C
##              cosmic_2
##           <character>
##      2523           T
##      5776           T
##     16739           T
##     18114           T
##     25195           A
##       ...         ...
##    973795           A
```

```
##    978788           T
##    980288           A
##   1009268           A
##   1019245           G
##   -------
##   seqinfo: 24 sequences from an unspecified genome; no seqlengths
```

The following function adds metadata from one GRanges object to another for any overlapping positions, while keeping all the positions in the query object.

```
addMetadata = function(query, subject, column.suffix) {

  # Find overlapping ranges
  hits = findOverlaps(query, subject)

  for ( column in names(mcols(subject)) ) {

    # Create empty metadata column to be filled
    mcols(query)[paste(column, column.suffix, sep='')] = NA

    # Convert DNAStringSet and DNAStringSetList columns to character vectors
    if (class(mcols(subject)[[column]])[1] == 'DNAStringSet') {
      mcols(subject)[column] = as.character(mcols(subject)[[column]])
    } else if (class(mcols(subject)[[column]])[1] == 'DNAStringSetList') {
      mcols(subject)[column] =
        unstrsplit(CharacterList(mcols(subject)[[column]]))
    }

    # Add subject metadata to query
    mcols(query)[queryHits(hits), paste(column, column.suffix, sep='')] =
      mcols(subject)[subjectHits(hits), column]
  }
  return(query)
}
```

## Comparison with COSMIC SNP profile

```
# Add COSMIC data to variant calling data.
data = as.data.frame(addMetadata(data.gr, cosmic.gr, ''))

# Filter and remove empty rows.
data = data[data$filter=='None' & data$DP > 9, ]

# Check for variant calling genotypes matching those in COSMIC.
alleles = c('cosmic_1', 'cosmic_2', 'sample_1', 'sample_2')
idx.notna = row.names(subset(data, rowSums(is.na(data[, alleles])) == 0))
data[idx.notna, 'match.cosmic'] = 'no'

data[idx.notna, 'for.1'] = paste(data[idx.notna, 'cosmic_1'],
                                 data[idx.notna, 'cosmic_2'], sep=':')
data[idx.notna, 'rev.1'] = paste(data[idx.notna, 'cosmic_2'],
                                 data[idx.notna, 'cosmic_1'], sep=':')
```

```
data['for.2'] = paste(data[, 'sample_1'], data[, 'sample_2'], sep=':')

idx.ok.1 = apply(data, 1, function(x) x['for.1'] %in% x['for.2'])
idx.ok.2 = apply(data, 1, function(x) x['rev.1'] %in% x['for.2'])
data[idx.ok.1, 'match.cosmic'] = 'yes'
data[idx.ok.2, 'match.cosmic'] = 'yes'
data$for.1 = NULL
data$rev.1 = NULL
```

## Comparison with Yu *et al.* SNP genotyping panel

The file *yu.snps.txt* is included in the R Markdown directory, but can also be found in the supplementary data of (Yu et al. 2015). RefSNP orientation data for the 48 loci included in the Yu panel can be found at the dbSNP website, but is also included here as the *refsnp.strand.txt* file. (Sherry et al. 2000)

```
# Load Yu data
yu = read.table('yu.snps.txt', header=TRUE, sep='\t')
yu[1:6, 1:5]
```

```
##     CLID    cName Cell.Line.Name rs10018359 rs10834627
## 1 587114    105KC          105KC        T:T        C:C
## 2 586793   1321N1         1321N1        C:T        T:T
## 3 584976     143B           143B        C:C        T:T
## 4 586577    184A1          184A1        C:T        C:C
## 5 584462    22Rv1          22Rv1        C:T        T:T
## 6 586854 23132/87       23132/87        T:T        C:T
```

```
yu$cName = tolower(gsub('[-_. ]', '', yu$cName))
yu = t(subset(yu, cName=='colo205'))
yu = data.frame(alleles=yu[4:nrow(yu), ])
yu$rsID = row.names(yu)
yu = data.frame(yu[yu$alleles != '', ])
yu$alleles = as.character(yu$alleles)

# Load RefSNP orientation data and merge with Yu data.
strand = read.table('refsnp.strand.txt', header=TRUE)
head(strand)
```

```
##       rsID strand
## 1  rs12537      +
## 2  rs18579      +
## 3 rs260690      +
## 4 rs279844      +
## 5 rs315791      +
## 6 rs316598      +
```

```
yu = merge(yu, strand, by='rsID', all.x=TRUE)

# Complement reverse-strand mutations.
idx = row.names(subset(yu, strand=='-'))
yu[idx, 'alleles'] = chartr('ATCG','TAGC', yu[idx, 'alleles'])
```

```r
# Compare Yu SNPs with variant calling data.
suppressPackageStartupMessages(library(plyr))

yu$alleles.rev = reverse(yu$alleles)
yu$strand = NULL
names(yu) = c('rsID', 'yu.genotype', 'rev.yu')
data = join(data, yu, by='rsID', type='left')

alleles = c('for.2', 'yu.genotype', 'rev.yu')
idx.notna = row.names(subset(data, rowSums(is.na(data[, alleles])) == 0))
data[idx.notna, 'match.yu'] = 'no'

idx.ok.1 = apply(data, 1, function(x) x['yu.genotype'] %in% x['for.2'])
idx.ok.2 = apply(data, 1, function(x) x['rev.yu'] %in% x['for.2'])
data[idx.ok.1, 'match.yu'] = 'yes'
data[idx.ok.2, 'match.yu'] = 'yes'
data$for.2 = NULL
data$rev.yu = NULL
```

# Results

Calculate and print comparisons between variant calling data and COSMIC / Yu SNPs, respectively.

```r
data.cosmic = data[!is.na(data$match.cosmic), ]
n.cosmic = dim(data.cosmic)[1]
n.cosmic.match = dim(data.cosmic[data.cosmic$match.cosmic=='yes', ])[1]
conc.cosmic = round(n.cosmic.match / n.cosmic * 100, 1)

data.yu = data[!is.na(data$match.yu), ]
n.yu = dim(data.yu)[1]
n.yu.match = dim(data.yu[data.yu$match.yu=='yes', ])[1]

cat(paste('Unique SNPs\tCalls\tMatches\tConcordance\tYu calls\tYu matches\n',
          n.unique.snps, '\t\t', n.cosmic, '\t', n.cosmic.match, '\t\t',
          conc.cosmic, '\t', n.yu, '\t', n.yu.match, '\n', sep=''))
```

```
## Unique SNPs  Calls   Matches Concordance Yu calls    Yu matches
## 241      68  67      98.5    2   2
```

## Results: RNA variant calls vs. COSMIC SNP profiles

Data is provided in *auth.collected.txt*.

```r
data = read.table('auth.collected.txt', header=TRUE, sep='\t',
                  stringsAsFactors = FALSE, na='')
data$match.cosmic = factor(data$match.cosmic, levels=c('yes', 'no'))
data$match.yu = factor(data$match.yu, levels=c('yes', 'no'))

suppressPackageStartupMessages(library(dplyr))
suppressPackageStartupMessages(library(ggplot2))
```

```r
# Total filtered variant calls per cell line (Table 1)
total.calls = data %>% group_by(sample) %>% summarise(count=n())
total.calls
```

```
## Source: local data frame [8 x 2]
##
##    sample  count
##     (chr)  (int)
## 1 colo205  38777
## 2    dld1  72203
## 3 hct116a 177948
## 4 hct116b  44231
## 5   hct15  55195
## 6    hke3 670153
## 7    ht29  38456
## 8     rko 250042
```

```r
# Count unique SNPs in each cell line (Table 2)
lines = c('colo205', 'hct116', 'hct15', 'ht29', 'rko')
lines.count = data.frame()
for ( line in lines ) {
  cosmic = cosmic.all[cosmic.all$sample.name == line, ]

  # Remove duplicates
  cosmic$gene.name = gsub('_ENST\\d+', '', cosmic$gene.name)
  cosmic = cosmic[!duplicated(cosmic[, c('gene.name',
                                         'mutation.genome.position')]), ]
  cosmic = cosmic[grep('Substitution', cosmic$mutation.description), ]

  lines.count[line, 'count'] = dim(cosmic)[1]
}
lines.count
```

```
##         count
## colo205   241
## hct116   2428
## hct15    7649
## ht29      462
## rko      2676
```

```r
# Variant calls in COSMIC sites (Table 2)
cosmic = data[!is.na(data$mutation.id), ]
cosmic.calls = cosmic %>% group_by(sample) %>% summarise(count=n())
cosmic.calls
```

```
## Source: local data frame [8 x 2]
##
##    sample count
##     (chr) (int)
## 1 colo205    68
## 2    dld1  2239
## 3 hct116a  1122
```

```
## 4 hct116b  1003
## 5   hct15  3356
## 6    hke3  1379
## 7    ht29   145
## 8     rko  1112
```

```r
# Concordance with COSMIC (Table 2)
cosmic.conc = cosmic %>% group_by(sample, match.cosmic) %>%
  summarise(count=n()) %>% mutate(perc=round(count/sum(count) * 100, 1))
cosmic.conc
```

```
## Source: local data frame [16 x 4]
## Groups: sample [8]
##
##     sample match.cosmic count  perc
##      (chr)       (fctr) (int) (dbl)
## 1  colo205          yes    67  98.5
## 2  colo205           no     1   1.5
## 3     dld1          yes  2211  98.7
## 4     dld1           no    28   1.3
## 5  hct116a          yes  1094  97.5
## 6  hct116a           no    28   2.5
## 7  hct116b          yes   986  98.3
## 8  hct116b           no    17   1.7
## 9    hct15          yes  3331  99.3
## 10   hct15           no    25   0.7
## 11    hke3          yes  1326  96.2
## 12    hke3           no    53   3.8
## 13    ht29          yes   143  98.6
## 14    ht29           no     2   1.4
## 15     rko          yes  1073  96.5
## 16     rko           no    39   3.5
```

```r
# Variant calls in Yu sites (Table 2)
yu = data[!is.na(data$match.yu), ]
yu.calls = yu %>% group_by(sample, match.yu) %>% summarise(count=n())
yu.calls
```

```
## Source: local data frame [8 x 3]
## Groups: sample [?]
##
##    sample match.yu count
##     (chr)   (fctr) (int)
## 1 colo205      yes     2
## 2    dld1      yes     2
## 3 hct116a      yes     2
## 4 hct116b      yes     2
## 5   hct15      yes     2
## 6    hke3      yes     7
## 7    ht29      yes     2
## 8     rko      yes     2
```

```
# Characterisation plot: SNP impact (Figure 3A)
cosmic$impact = factor(cosmic$impact, levels=c('HIGH', 'MODERATE', 'LOW',
                                              'MODIFIER'))
impact = cosmic %>% group_by(match.cosmic, impact) %>% summarise(count=n()) %>%
  mutate(perc=round(count/sum(count) * 100, 1))
gg.impact = ggplot(impact, aes(x=impact, y=perc, fill=match.cosmic)) +
  geom_bar(stat='identity', position='dodge', color='black', size=0.3) +
  theme_bw() +
  labs(x='\nImpact', y='Proportion of calls (%)\n') +
  theme(legend.position='none', axis.text=element_text(size=14),
        panel.grid.major.x=element_blank(),
        axis.title=element_text(size=17.5)) +
  geom_text(data=impact, aes(label=count), position=position_dodge(width=0.9),
            vjust=-0.5) +
  scale_fill_grey()
gg.impact
```

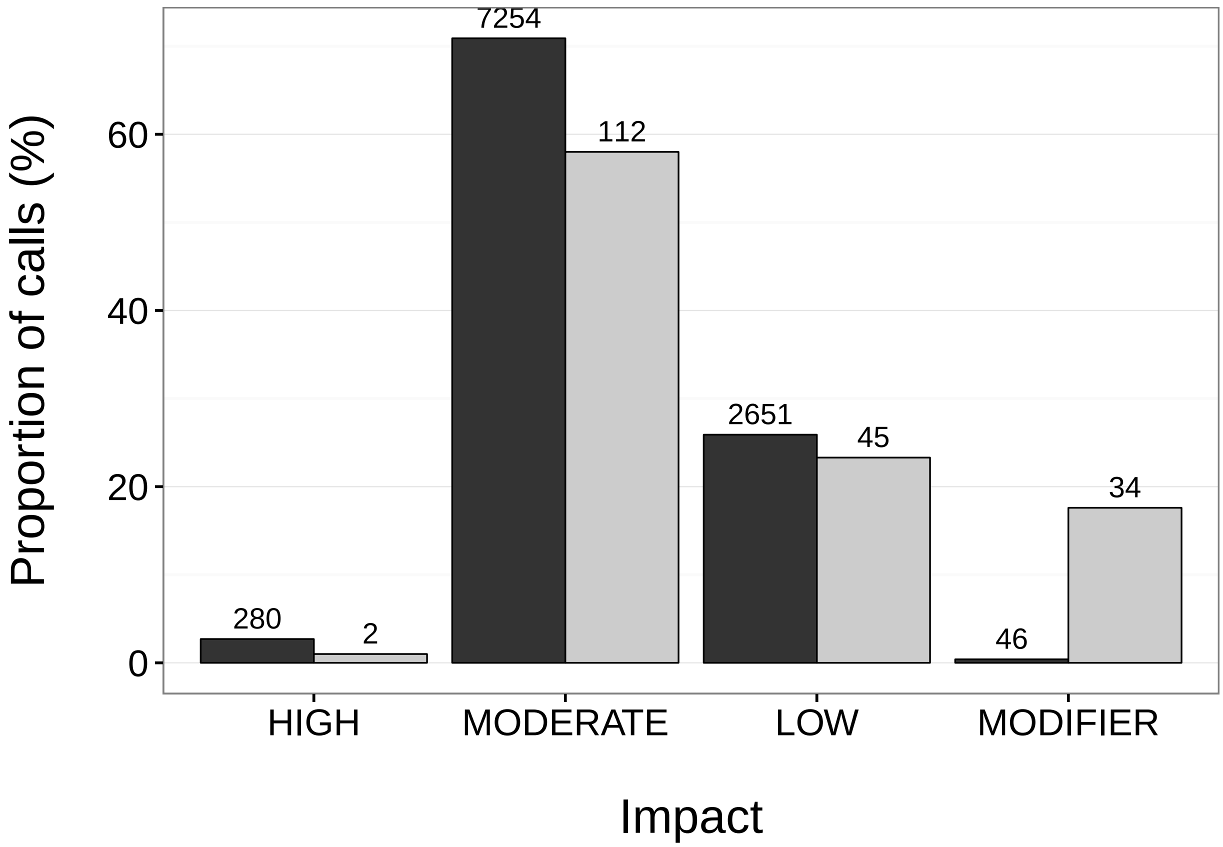


```
# Characterisation plot: SNP type (Figure 3B)
cosmic$mutation.description = gsub('Substitution - ', '',
                                   cosmic$mutation.description)
cosmic$mutation.description = factor(cosmic$mutation.description,
  levels=c('Missense', 'Nonsense', 'coding silent'))
type = cosmic %>% group_by(match.cosmic, mutation.description) %>%
  summarise(count=n()) %>% mutate(perc=round(count/sum(count)*100, 1))
gg.type = ggplot(type, aes(x=mutation.description, y=perc, fill=match.cosmic)) +
  geom_bar(stat='identity', position='dodge', color='black', size=0.3) +
```

```
  theme_bw() +
  labs(x='\nSNP type', y='Proportion of calls (%)\n') +
  theme(legend.position='none', axis.text=element_text(size=14),
        panel.grid.major.x=element_blank(),
        axis.title=element_text(size=17.5)) +
  scale_x_discrete(labels=c('Missense', 'Nonsense', 'Coding silent')) +
  geom_text(data=type, aes(label=count), position=position_dodge(width=0.9),
            vjust=-0.5) +
  scale_fill_grey()
gg.type
```

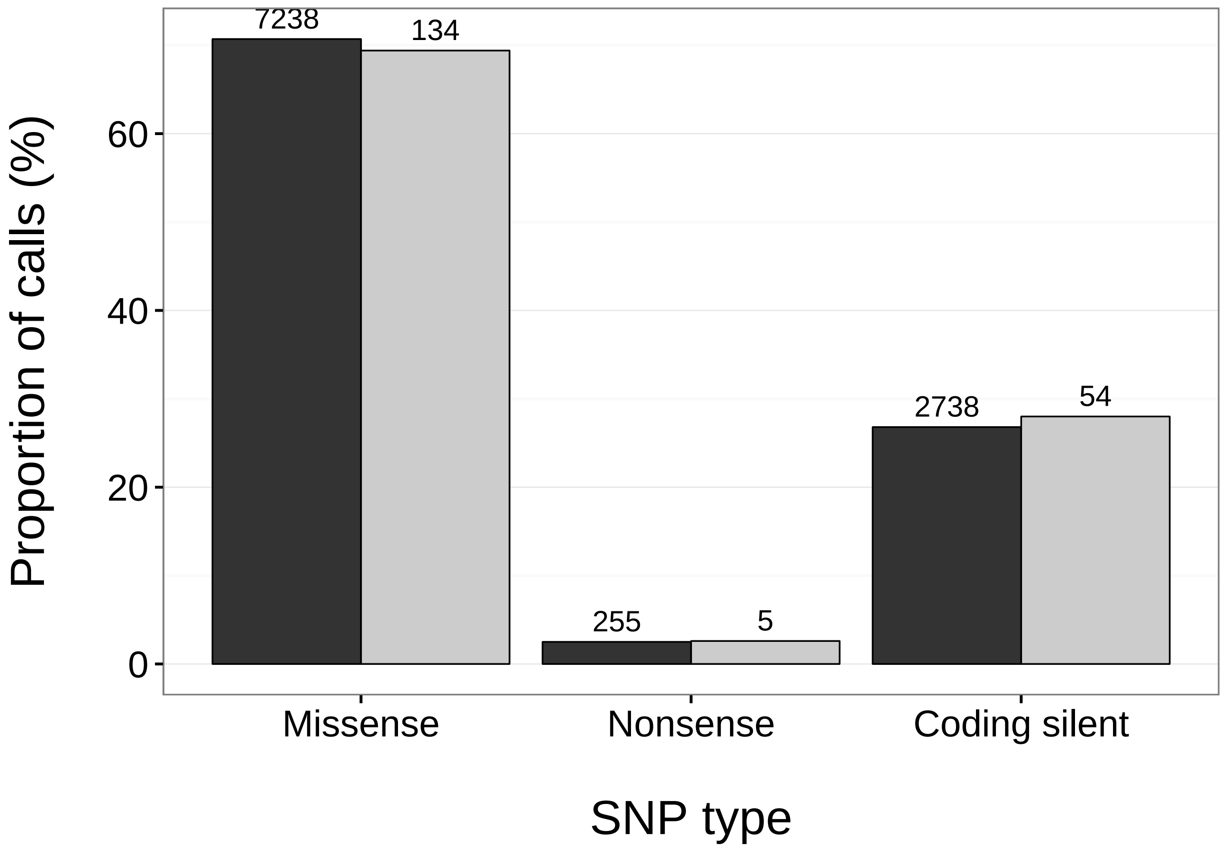


### Results: Subsamples vs. COSMIC SNP profiles

Data is provided in *auth.subsamples.collected.txt*.

```
subsamples = read.table('auth.subsamples.collected.txt', header=TRUE, sep='\t',
                        stringsAsFactors=FALSE, na='')

# Subset for COSMIC data; get cell line names and subsampling proportions.
cosmic = subsamples[!is.na(subsamples$match.cosmic), ]
cosmic$name = sapply(cosmic$sample, function(x) strsplit(x, '_')[[1]][1])
cosmic$subsample = sapply(cosmic$sample, function(x) strsplit(x, '_')[[1]][3])

# Get total and matching number of COSMIC SNPs per cell line.
data = cosmic %>% group_by(name, subsample) %>% summarise(total=n())
```

```
matches = cosmic[cosmic$match.cosmic=='yes', ] %>%
  group_by(name, subsample) %>% summarise(yes=n())

# Merge, calculate concordance, prepare for plotting.
data$yes = matches$yes
data$conc = round(data$yes / data$total * 100, 1)
data$subsample = as.numeric(data$subsample) * 100
data$subsample = factor(data$subsample, levels=c('100', '90','80', '70',
  '60', '50', '40', '30', '20', '10', '5', '2', '1'))
cbPalette = c("#0072B2", "#E69F00", "#56B4E9", '#ECDE13', "#009E73", "#D55E00")

# Subsampling plot: HCT116a only (Figure 2)
hct116a = data[data$name=='hct116a', ]
hjust = c(rep(1.15, 7), rep(0.75, 2), 0.65, rep(0.5, 3))
vjust = c(rep(1.5, 2), rep(1, 5), rep(2, 6))

gg.hct116a = ggplot(hct116a, aes(x=subsample, y=total)) +
  geom_point(size=2, colour='black') +
  geom_line(data=hct116a, aes(group=1), colour='black') +
  theme_bw() +
  labs(x='\nSubsampling proportion (%)', y='Number of COSMIC SNPs\n') +
  theme(legend.position='none') +
  geom_text(data=hct116a, aes(label=paste(conc, '%', sep='')), vjust=vjust,
            hjust=hjust, colour='black')
gg.hct116a
```

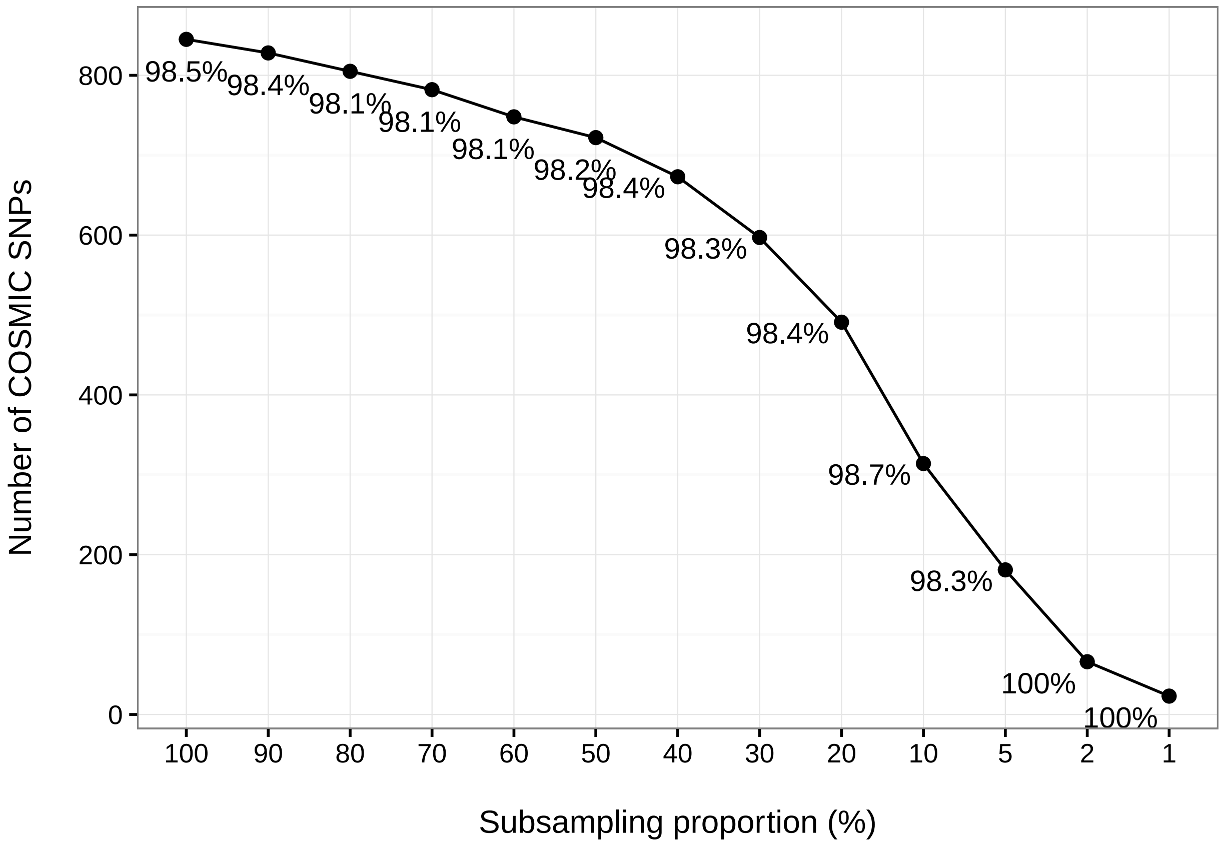

```
# Subsampling plot: number of COSMIC SNPs (Figure S2)
gg.snps = ggplot(data, aes(x=subsample, y=total, colour=name)) +
  geom_point() +
  geom_line(data=data, aes(group=name)) +
  theme_bw() +
  labs(x='\nSubsampling proportion (%)', y='Number of COSMIC SNPs\n') +
  scale_colour_manual(name='Cell line', values=cbPalette) +
  theme(legend.position='none')
gg.snps
```

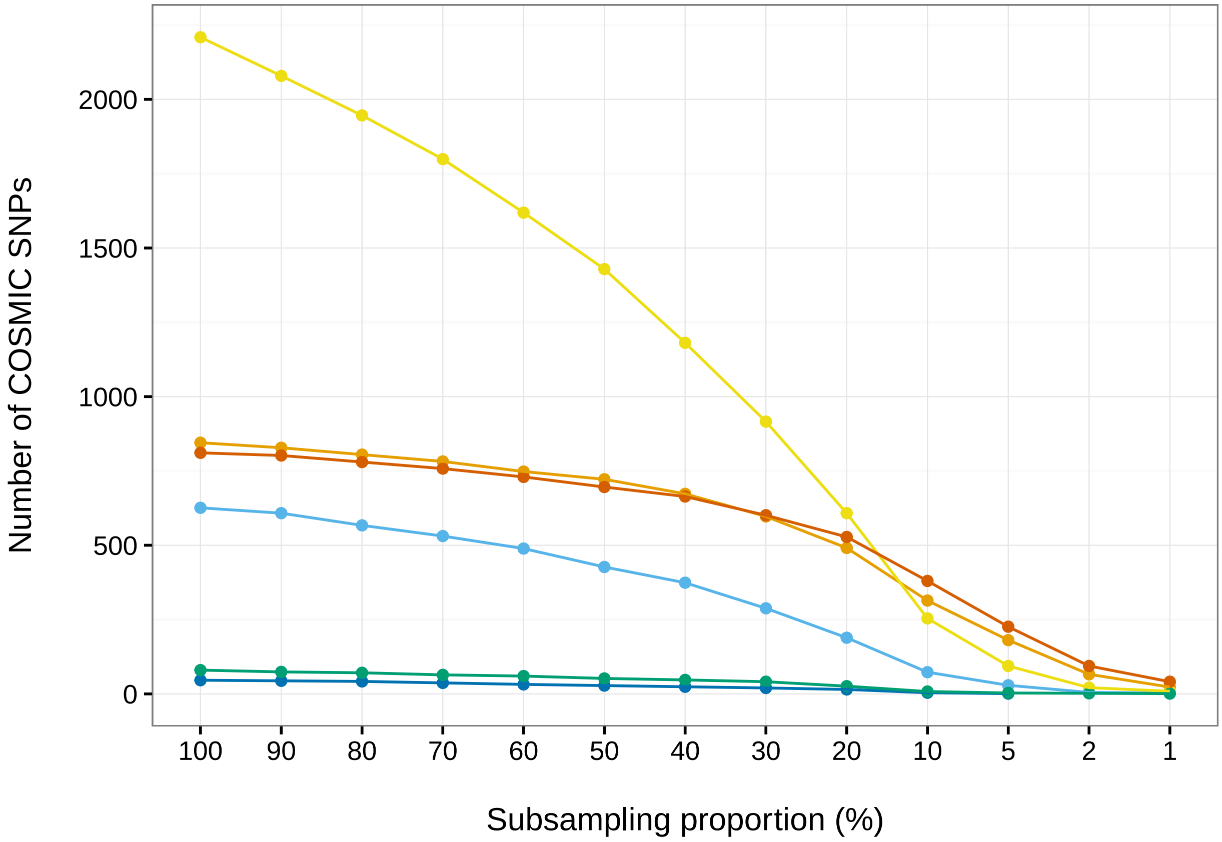


```
# Subsampling plot: concordance (Figure S2)
gg.conc = ggplot(data, aes(x=subsample, y=conc, colour=name)) +
  geom_point() +
  geom_line(data=data, aes(group=name)) +
  theme_bw() +
  labs(x='\nSubsampling proportion (%)', y='Concordance (%)\n') +
  scale_colour_manual(name='Cell line', values=cbPalette) +
  theme(legend.position='none') +
  ylim(90, 100)
gg.conc
```

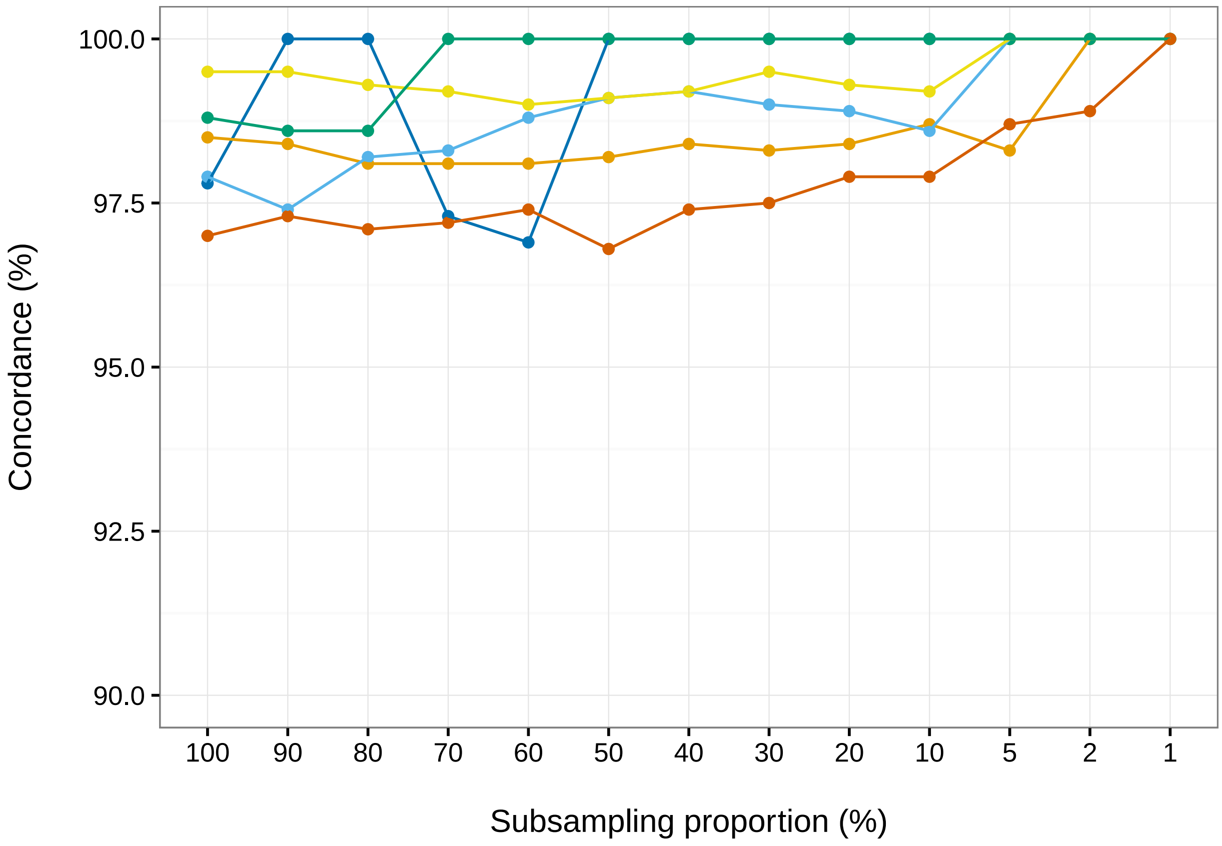


### Results: All RNA SNPs vs. all RNA SNPs

Data is provided in *auth.rna.vs.rna.collected.txt*.

```
# Load data
data.all.rna = read.table('auth.rna.vs.rna.collected.txt',
                              header=TRUE, sep='\t', na='', fill=TRUE)

# Create data frame to be filled out with data
lines = c('colo205', 'dld1', 'hct116a', 'hct116b',
          'hct15', 'hke3', 'ht29', 'rko')
data = data.frame()
n = 1
for ( line in lines ) {
  for ( comp in lines ) {
    data[n, 'sample'] = line
    data[n, 'comparison'] = comp
    n = n + 1
  }
}

# Add total SNPs
total = data.all.rna %>% group_by(sample.input.1, sample.input.2) %>%
  summarise(total=n())
total = total[!is.na(total$sample.input.2), ]
data = merge(data, total, by.x=c('sample','comparison'),
```

```
             by.y=c('sample.input.1','sample.input.2'), all.x=TRUE)

# Add concordance
yes = data.all.rna %>% group_by(sample.input.1, sample.input.2, match) %>%
  summarise(matches=n())
yes = yes[!is.na(yes$sample.input.2), ]
yes = yes[yes$match=='yes', ]
data = merge(data, yes[c(1:2, 4)], by.x=c('sample','comparison'),
             by.y=c('sample.input.1','sample.input.2'), all.x=TRUE)
data$concordance = round(data$matches / data$total * 100, 1)

# Plot
data$text.colour = 'black'
data[data$concordance > 90, 'text.colour'] = 'white'
data$sample = toupper(data$sample)
data$comparison = toupper(data$comparison)
data[data$sample=='HCT116A', 'sample'] = 'HCT116a'
data[data$sample=='HCT116B', 'sample'] = 'HCT116b'
data[data$comparison=='HCT116A', 'comparison'] = 'HCT116a'
data[data$comparison=='HCT116B', 'comparison'] = 'HCT116b'
gg.all.rna = ggplot(data, aes(x=sample, y=comparison, fill=concordance)) +
  geom_tile(colour='black', size=0.3) +
  coord_equal() +
  theme(axis.ticks=element_blank(), panel.background=element_blank(),
        axis.text.x=element_text(angle=90, hjust=1)) +
  labs(x=NULL, y=NULL, fill='Concordance (%)') +
  geom_text(colour=data$text.colour, size=3, aes(
    label=paste(concordance, ' %', sep=''))) +
  scale_fill_gradient(low='white', high='#666666')
gg.all.rna
```

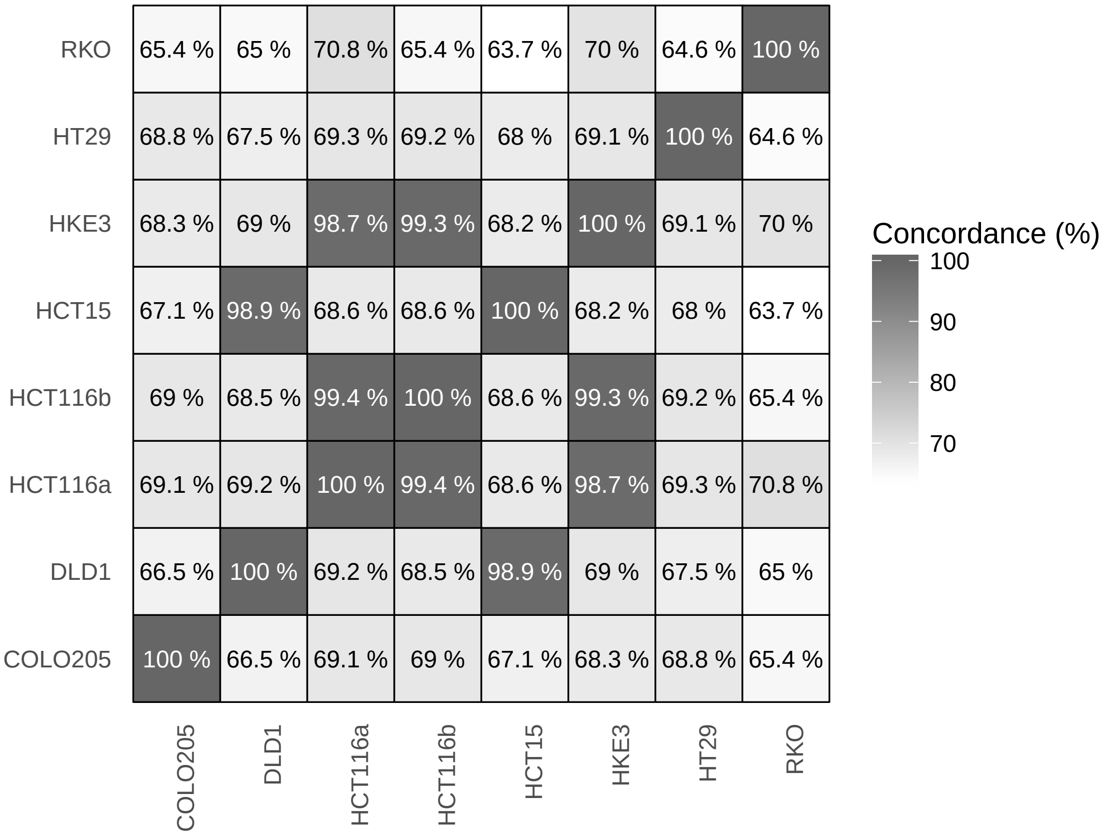


## Results: All RNA SNPs vs. COSMIC SNP profiles

Data is provided in *auth.rna.vs.cosmic.collected.txt*.

```
# Load data
data.all.cosmic = read.table('auth.rna.vs.cosmic.collected.txt',
                             header=TRUE, sep='\t')

# Create data frame to be filled out with data
lines = c('colo205', 'dld1', 'hct116a', 'hct116b',
          'hct15', 'hke3', 'ht29', 'rko')
comparisons = c('colo205', 'hct116', 'hct15', 'ht29', 'rko')
data = data.frame()
n = 1
for ( line in lines ) {
  for ( comp in comparisons ) {
    data[n, 'sample'] = line
    data[n, 'comparison'] = comp
    n = n + 1
  }
}

# Add totals and unique SNPs
total = data.all.cosmic %>% group_by(sample.name, sample) %>%
  summarise(total=n())
data = merge(data, total, by.x=c('sample','comparison'),
```

```r
             by.y=c('sample','sample.name'), all.x=TRUE)
data = merge(data, lines.count, by.x='comparison', by.y='row.names', all.x=TRUE)
data$coverage = round(data$total / data$count * 100, 1)

# Add concordance
yes = data.all.cosmic %>% group_by(sample.name, sample, match.cosmic) %>%
  summarise(yes=n())
yes = yes[yes$match.cosmic=='yes', ]
yes$match.cosmic = NULL
data = merge(data, yes, by.x=c('sample','comparison'),
             by.y=c('sample','sample.name'), all.x=TRUE)
data$concordance = round(data$yes / data$total * 100, 1)
data[is.na(data)] = 0

# Plot
data$text.colour = 'black'
data[data$coverage > 20, 'text.colour'] = 'white'
data$sample = toupper(data$sample)
data$comparison = toupper(data$comparison)
data[data$sample=='HCT116A', 'sample'] = 'HCT116a'
data[data$sample=='HCT116B', 'sample'] = 'HCT116b'
data$sample = factor(data$sample,
  levels=c('COLO205','HCT116a','HCT116b','HKE3','DLD1','HCT15','HT29','RKO'))

gg.all.cosmic = ggplot(data, aes(x=sample, y=comparison, fill=coverage)) +
  geom_tile(colour='black', size=0.3) +
  coord_equal() +
  theme(axis.ticks=element_blank(), panel.background=element_blank(),
        axis.text.x=element_text(angle=90, hjust=1)) +
  labs(x='\nRNA-seq variant calls', y='COSMIC profile', fill='Coverage (%)') +
  scale_fill_gradient(low='white', high='#666666') +
  geom_text(color=data$text.colour, size=3,
            aes(label=paste(concordance, '%\n(', coverage, '%)', sep='')))
gg.all.cosmic
```

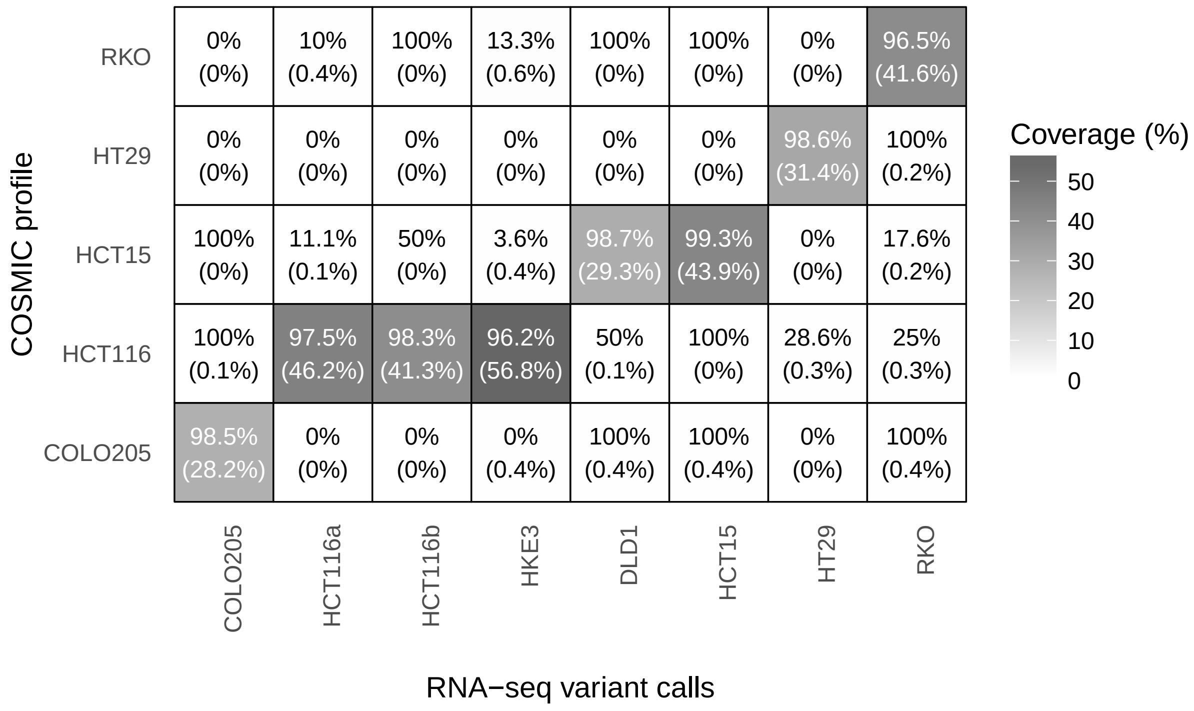


## Session info

```
## R version 3.2.3 (2015-12-10)
## Platform: x86_64-apple-darwin13.4.0 (64-bit)
## Running under: OS X 10.10.5 (Yosemite)
##
## locale:
## [1] en_US.UTF-8/en_US.UTF-8/en_US.UTF-8/C/en_US.UTF-8/en_US.UTF-8
##
## attached base packages:
## [1] stats4    parallel  stats     graphics  grDevices utils     datasets
## [8] methods   base
##
## other attached packages:
## [1] ggplot2_2.0.0        dplyr_0.4.3          plyr_1.8.3
## [4] GenomicRanges_1.22.4 GenomeInfoDb_1.6.3   IRanges_2.4.6
## [7] S4Vectors_0.8.11     BiocGenerics_0.16.1
##
## loaded via a namespace (and not attached):
##  [1] Rcpp_0.12.3      knitr_1.12.3     XVector_0.10.0   magrittr_1.5
##  [5] zlibbioc_1.16.0  munsell_0.4.2    colorspace_1.2-6 R6_2.1.2
##  [9] stringr_1.0.0    tools_3.2.3      grid_3.2.3       gtable_0.1.2
## [13] DBI_0.3.1        htmltools_0.3    lazyeval_0.1.10  yaml_2.1.13
## [17] digest_0.6.9     assertthat_0.1   formatR_1.3      evaluate_0.8
## [21] rmarkdown_0.9.5  labeling_0.3     stringi_1.0-1    scales_0.3.0
```

# References

Forbes, Simon A, David Beare, Prasad Gunasekaran, Kenric Leung, Nidhi Bindal, Harry Boutselakis, Minjie Ding, et al. 2015. "COSMIC: exploring the world's knowledge of somatic mutations in human cancer." *Nucleic Acids Research* 43 (Database issue): D805–11.

Sherry, S T, M H Ward, M Kholodov, J Baker, L Phan, E M Smigielski, and K Sirotkin. 2000. "dbSNP: the NCBI database of genetic variation." *Nucleic Acids Research* 29 (1): 308–11.

Yu, Mamie, Suresh K Selvaraj, May M Y Liang-Chu, Sahar Aghajani, Matthew Busse, Jean Yuan, Genee Lee, et al. 2015. "A resource for cell line authentication, annotation and quality control." *Nature* 520 (7547): 307–11.
